# Reactivity of a Sodium Anion with Alkenes: 1‑Electron vs 2‑Electron Reductions

**DOI:** 10.1021/acs.inorgchem.5c04279

**Published:** 2025-12-12

**Authors:** Huanxin Zhang, Nathan Davison, Jack M. Hemingway, Louise Male, Paul G. Waddell, Joshua Deakin, Floriana Tuna, James A. Dawson, Erli Lu

**Affiliations:** † School of Chemistry, 1724 University of Birmingham, Edgbaston, Birmingham B15 2TT, U.K.; ‡ Chemistry−School of Natural and Environmental Sciences, 5994 Newcastle University, Newcastle upon Tyne NE1 7RU, U.K.; § Department of Chemistry and Photon Science Institute, 5292 University of Manchester, Oxford Road, Manchester M13 9PL, U.K.

## Abstract

The sodium anion (sodide) is an uncommon alkali metal species, whose reactivity has been scarcely studied, with previous reports limited to decomposition and quenching reactions of ill-characterized species formed in situ. Based on our recent report of the first gram-scale synthesis of a well-characterized sodide complex, [Na^+^(2,2,2-cryptand)]­Na^–^ (**1**), we now describe its reactivity with phenyl-substituted ethylenes and a cyclic polyolefin. Depending on the substitution pattern, **1** mediates either 1-electron or 2-electron reduction. Reaction with a cyclic multiolefin, 1,3,5,7-cyclooctatetraene (COT), exhibits two-electron reduction to produce COT^2–^ species.

## Graphical abstract

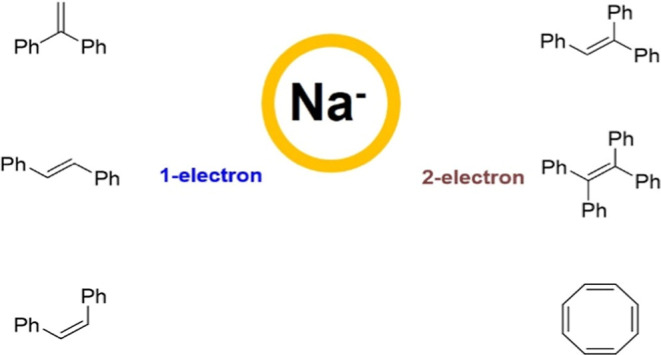

## Introduction

The alkali metals (Li–Cs) are some of the most abundant, low-cost elements of all the periodic table. The chemistry of the alkali metals is dominated by the +1 oxidation state. Although chemical reduction of alkali metal cations to their corresponding zerovalent metal was historically rare,^1^ AM^+^ → AM^0^ reduction has been increasingly reported recently.^2–7^ Beyond this, complexes containing alkali metal anions (alkali metals in the −1 oxidation state, _*n*_S^2^) are known as alkalides.^8^

To date, low-oxidation state s-block metal chemistry is dominated by the alkaline earth metals, since the first magnesium­(I) dimers were reported by Cameron Jones in 2007.^9^ While molecular zerovalent alkali metal complexes remain unknown, molecular low-oxidation state group-1 chemistry has been documented since 1974 when James L. Dye reported the first isolated alkali metal anion complex, [Na^+^(2,2,2-cryptand)]­Na^–^ (**1**), containing a Na^–^ anion.^10,11^ In the following years, James L. Dye overcame a number of challenging synthetic hurdles to isolate an impressive range of alkalides (Na–Cs).^8^ However, despite their huge potential as soluble, stoichiometric, and controllable reducing agents, their reactivity is severely underdeveloped and underutilized.

The study of alkalides has been significantly constrained due to their very challenging synthesis and handling requirements. As a result, almost all the previous reported reactivity studies involve the in situ formation of the alkalide species, rather than from isolated, well-characterized complexes. The isolated products from these studies are usually quenched organic products,^12–16^ rather than organometallic products that would allow us to gain a more in-depth understanding of how alkalides react toward organic substrates.

Moreover, there are two major disadvantages that arise from in situ approaches: (1) the reactive species formed in situ is not always clear, since the formation of alkalides and electrides compete and exist in equilibrium with each other.^17^ (2) The in situ formation of the alkalide species in solution will compete with decomposition of the alkalide at room temperature. Put together, these factors complicate the reaction picture and could result in reproducibility issues.

These difficulties originate from the synthetic hurdles of alkalides, including the requirements of specialized, bespoke glassware, low boiling point solvents (such as dimethyl ether), distilled high-purity alkali metals, vigorously clean glassware (requiring treatment with HF/HNO_3_), in some cases, metal-vapor deposition apparatus, and the decomposition of some of the alkalides at room temperature once formed.^18–20^ As a result, it was impossible to obtain alkalides in a bulk, synthetically meaningful amount to allow a proper reactivity study. Indeed, in all of the alkalide reports prior to 2024, the product yield was rarely reported.

In 2024, we reported the first scalable synthesis of the archetypical alkalide, a sodide complex [Na^+^(2,2,2-cryptand)]­Na^–^ (**1**), at gram-scale, by using mechanochemistry to directly react sodium metal and 2,2,2-cryptand solvent-free.^21^ As a cheap, Earth abundant, environmentally benign metal, there has been much progress and interest in recent years of using sodium metal as a reagent, for example, in the degradation of polytetrafluoroethylene (PTFE)^22,23^ and the direct synthesis of organosodium compounds.^24–29^

This advance allows synthetic chemists to access the sodium anion as a discrete reagent for controlled reactivity studies. As far as the authors are aware, the only case of alkalide reactivity using a crystallized alkalide complex, prior to our report in 2024, was by Dye and co-workers who reacted a crystalline caeside with ethylene (solid–gas reaction), kept at −40 °C for 1 week, to form ethane and butane, although the source of the additional protons was unable to be determined.^30^

Alkalides can act as one- or two-electron reductants, although at present there is a current knowledge gap in understanding under what conditions a 1- or 2-electron reduction may take place, due to the lack of any current systematic reactivity study. Moreover, in contrast to zerovalent alkali metals, alkalides (AM^–^) can conduct single-metal-two-electron reductions, allowing the alkalides to potentially undergo transition-metal-like 2-electron redox processes.

Building on the observation that alkalides can reduce the unsaturated bond in ethylene, we aimed at undertaking a systematic reactivity study of a sodide complex toward C=C bonds, selecting phenyl-substituted ethylenes and cyclooctatetraene (COT) as representative substrates, isolating the organometallic intermediates, and uncovering substitution-dependent divergences between 1-electron and 2-electron reduction outcomes.

## Results and Discussion

The reaction between **1** and styrene led to an intractable mixture. While ^1^H NMR and ^23^Na NMR spectra showed that styrene and **1** were all consumed, there was no dissolved Na-containing species in the product mixture (Figures S23 and S24). Gratifyingly, the reaction of **1** with 1 equiv of 1,1-diphenylethylene produced complex **2** in 73% yield as a red crystalline solid (Figure 1a). The solid-state molecular structure of **2** was unveiled by the SCXRD study to be the separated ion pair (SIP) complex [Na^+^(2,2,2-cryptand)]_2_[Ph_2_CCH_2_CH_2_CPh_2_]^2–^, as shown in Figure 2a. The Ph_2_ *C*–*C*H_2_ (*C*_37_–*C*_50_ 1.5240(18) Å, *C*_52_–*C*_51_ 1.5263(17) Å, and *C*H_2_–*C*H_2_ (*C*_50_–*C*_51_ 1.5457(18) Å) bond lengths are typical of a carbon–carbon single bond (cf. 1.511(5) Å for Ph_2_CH*C*H_2_–*C*H_2_CHPh_2_ bond and 1.522(3) Å for Ph_2_ *C*H–*C*H_2_CH_2_CHPh_2_ bond^31^). These bond lengths in **2** are also close to the bond lengths of the previously reported polymeric complex [(Ph_2_CCH_2_CH_2_CPh_2_)­Na_2_(THF)_3_]_∞_ ^32^ by Izod and co-workers, in which the Na^+^ coordinated with a phenyl ring and one or two THF molecules, (Ph_2_C–CH_2_ (1.5232(19) Å) and CH_2_–CH_2_ (1.549(3) Å).

**Figure 1:**
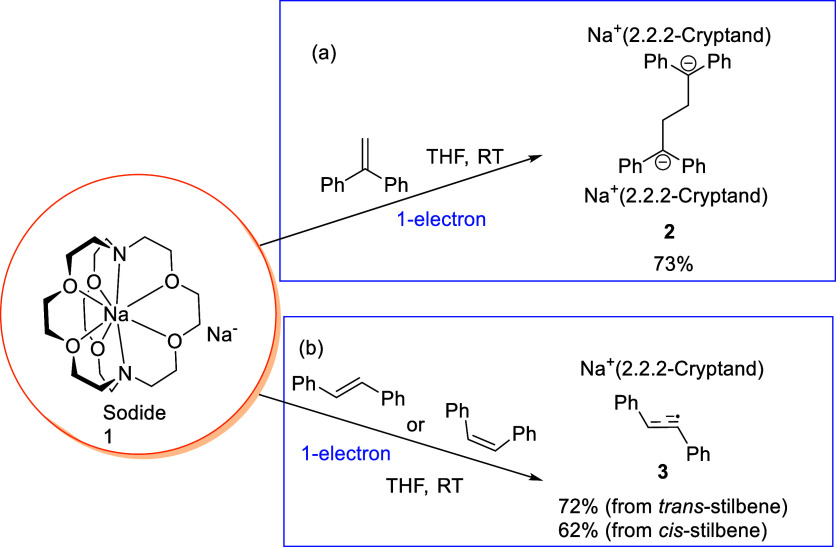
Reactions between the sodide **1** with (a) 1,1-diphenylethylene; (b) *cis*- and *trans*-stilbenes.

**Figure 2:**
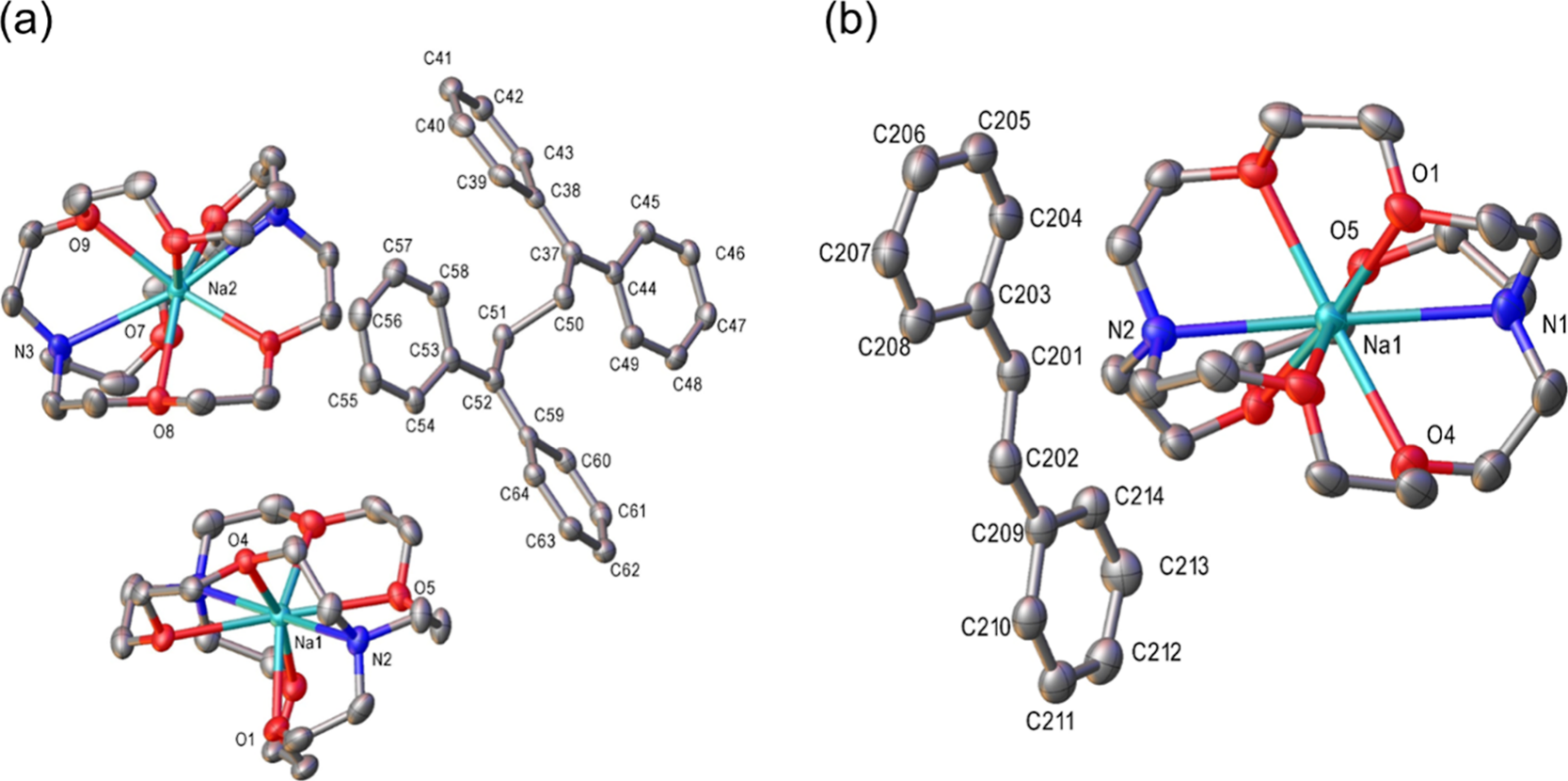
(a) Crystal structure of [Na^+^(2,2,2-cryptand)]_2_[Ph_2_CCH_2_CH_2_CPh_2_]^2–^ (**2)** at 50% thermal ellipsoid. Key bond lengths (Å): C37–C38 1.4340(19), C37–C44 1.4393(19), C38–C39 1.432(2), C38–C43 1.4338(19), C39–C40 1.379(2), C40–C41 1.390(2), C41–C42 1.392(2), C42–C43 1.380(2), C50–C51 1.5457(18), C51–C52 1.5263(17), C52–C53 1.4408(19), C52–C59 1.4258(19). Key bond angles (°): C38–C37–C44 125.54(12), C38–C37–C50 117.37(11), C44–C37–C50 117.03(12), C39–C38–C37 121.71(12),C39–C38–C43 112.55(12), C43–C38–C37 125.64(12), C37–C50–C51 115.59(10), C52–C51–C50 116.58(10), C53–C52–C51 117.16(12), C59–C52–C51 118.71(12), C59–C52–C53 124.11­(11). Hydrogen atoms are omitted for clarity; (b) Crystal structure of [Na^+^(2,2,2-cryptand)]­[PhCH–CHPh]^•–^ (**3)** at 50% thermal ellipsoid. Key bond lengths (Å): C(201)–C(202) 1.385(5), C(201)–C(203) 1.415(7), C(202)–C(209) 1.423(5), C(203)–C(204) 1.419(9), C(203)–C(208) 1.433(6), C(204)–C(205) 1.376(9), C(205)–C(206) 1.380(9), C(206)–C(207) 1.398(8), C(207)–C(208) 1.390(6). Key bond angles (°): C(202)–C(201)–C(203) 127.8(4), C(201)–C(202)–C(209) 126.6(4), C(201)–C(203)–C(204) 121.1(6), C(201)–C(203)–C(208) 124.0(5), C(204)–C(203)–C(208) 114.9(6), C(205)–C(204)–C(203) 122.0(8), C(204)–C(205)–C(206) 122.7(7), C(205)–C(206)–C(207) 117.3­(6), C(208)–C(207)–C(206) 121.4(6), C(207)–C(208)–C(203) 121.8(6), C(202)–C(209)–C(214) 122.8(5), C(210)–C(209)–C(202) 121.9(4), C(210)–C(209)–C(214) 115.2(4). The structure contains two crystallographically independent Na­(Cryptand) and stilbene molecules per asymmetric unit, of which only one is shown for clarity. Hydrogen atoms are omitted and only the disordered component with the highest occupancy is shown for clarity.

Once crystallized, **2** was poorly soluble in aromatic, aliphatic, and ethereal solvents and reacts with halogenated solvents, which prevented its NMR characterization. Additionally, **2** was exceedingly air- and moisture-sensitive, which prevented its CHN elemental analysis. The bulk product of **2** was tested several times with a single-crystal X-ray diffraction cell check. Additionally, we ran powder X-ray diffraction (PXRD) of the bulk crystalline solid which matched the simulated pattern from the SCXRD data, giving us confidence about its bulk purity.

Compound **2** is a result of Schlenk dimerization of the singly reduced 1,1-diphenylethyl anionic radical, which is well-established for zerovalent alkali metals as reductants^32,33^ and is different from the reported reactivity of a Mg­(I) dimer by Jones and co-workers, in which 1,1-diphenylethylene inserted into the Mg­(I)–Mg­(I) bond.^34^ Here, the Na^–^ anion center acts as an 1-electron reductant, while we postulate that the resultant Na^0^ species aggregates into Na metal particles and precipitates out from the THF solution, as gray precipitate was observed in the reaction mixture. Similar precipitate of zerovalent alkali metal particles was observed by Hill and co-workers recently in a report of alkali metal cation reduction reactions.^3^

In order to gain an understanding of the electronic structure and charge distribution of **2**, DFT calculations were performed. The negative charge of the dianion is found to be delocalized around the entire organic component (NPA charges, as shown in Figure S31a), with the largest NPA charge build up (−0.391 e average) located on the central carbon atoms with also some negative charge build up on the *ortho*- and *para*-sites of the four phenyl rings (−0.263 and −0.336 e, respectively).

To further explore the reactivity, **1** was treated with 1 equiv of *cis*- or *trans*-stilbene in THF solution. During both reactions, a rapid color change was observed from deep blue (the color of the sodide solution) to deep brown, with both reactions producing the radical anion complex [Na^+^(2,2,2-cryptand)]­[PhCH–CHPh]^•‑^ (**3**), which is a result of an 1-electron reduction of stilbene (Figure 1b). **3** was characterized by ^1^H and ^23^Na NMR spectroscopy, SCXRD (Figure 2b), PXRD, UV/vis absorption spectroscopy, and electron paramagnetic resonance (EPR) (see Supporting Information for details). The key structural feature of **3** is its monoanionic radical: the X-band EPR signal (from a dilute THF solution) exhibits well-resolved hyperfine couplings with neighboring 6 types of ^1^H nuclei, which is corroborated by EPR simulations and previous work from Okazaki and co-workers^35^ (Figures 3a and S6).

**Figure 3:**
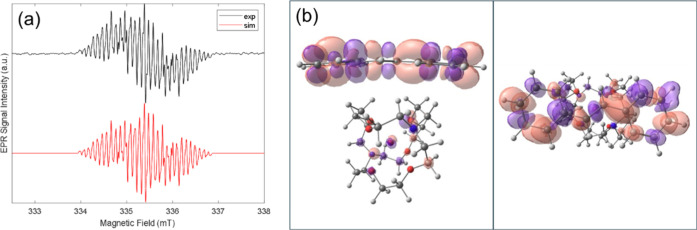
(a) X-band EPR spectra of **3** dilute sample in THF at room temperature (black), along with its simulated version (red). (*g* = 2.0024, proton hyperfine coupling constraints (in mT): 0.432, 0.389, 0.296, 0.19, 0.085, 0.0295 (2H, 2H, 2H, 2H, 2H, 2H)); (b) diagrams of the spin density orbitals of **3** from both a side view (left) and a top view (right), images generated using Chemcraft 1.8 with a contour value of 0.004.

Green single crystals of **3** suitable for the SCXRD study resulted from a THF/*n*-hexane solution at room temperature. The SCXRD structure of the stilbene monoanionic radical (Figure 2b) is nearly planar and features a *C*H=*C*H bond length of 1.378(11) Å, which is slightly longer than a typical double bond (cf. 1.310(7) Å in a cocrystallized *trans*-stilbene^36^). The ^1^H NMR spectrum of **3** exhibits broad signals for the 2,2,2-cryptand that encapsulates the sodium cation; however, the C*H*s and phenyls of the reduced anionic stilbene fragment are absent. This observation is in accordance with its SIP structure and the radical nature of the anion.

NPA charge analysis confirmed that the total charge over the radical anion component was −0.959 e, with the charge delocalized across the system with most of the negative character being present on the central, *ortho*- and *para*-carbons (−0.325, −0.267 and −0.311 e, respectively—Figure S31b). Moreover, the spin density calculation of **3** shows that the electron density spin is delocalized across the [PhCHCHPh]^•–^ fragment (Figure 3b), which is in accordance with the EPR signal. The observation that Na^–^ reacts with stilbenes giving **3** via an 1-electron reduction is in sharp contrast with Li or Na metal, where a 1,2-dianion [PhCHCHPh]^2–^ was generated as a result of a 2-electron reduction.^37^

Next, we treated **1** with one equivalent of triphenylethylene in THF which resulted in a very rapid color change of the reaction mixture from deep blue to reddish-purple. The product, [Na^+^(2,2,2-cryptand)]­[Ph_2_C–CH_2_Ph]^−^ (**4**), was obtained after crystallization as a greenish-black crystalline solid in 66% yield (Figure 4a). **4** was comprehensively characterized by NMR spectroscopy (^1^H, ^13^C, ^23^Na, DEPT135, ^1^H–^13^C HSQC), UV/vis absorption spectroscopy, SCXRD, and PXRD (see Supporting Information for details). **4** features a [Ph_2_C–CH_2_Ph]^1–^ monoanion, which is unambiguously confirmed by its SCXRD structures (Figure 5a) and NPA charge calculations (Figure S31c). The bond angle of C33–C32–C19 is 115.7(2)°, which is close to the similar skeleton (cf. 115.859°) in 1,1,1,2-tetraphenylethane.^38^ The ^1^H NMR spectra shows a single peak at 3.70 ppm corresponding to C*H*_*2*_Ph. We postulate the formation of **4** results from the protonation of a two-electron reduced 1,2-dicarbanion (Figure 4a) intermediate, where the proton source was unable to be identified. To further prove the bulky purity, we also ran PXRD for this sample, which gave the same pattern simulated from SCXRD data. 1,2-dicarbanions are a highly reactive and scarce species, with only few examples of their organo-alkali metal complexes reported over the decades.^37,39^

**Figure 4:**
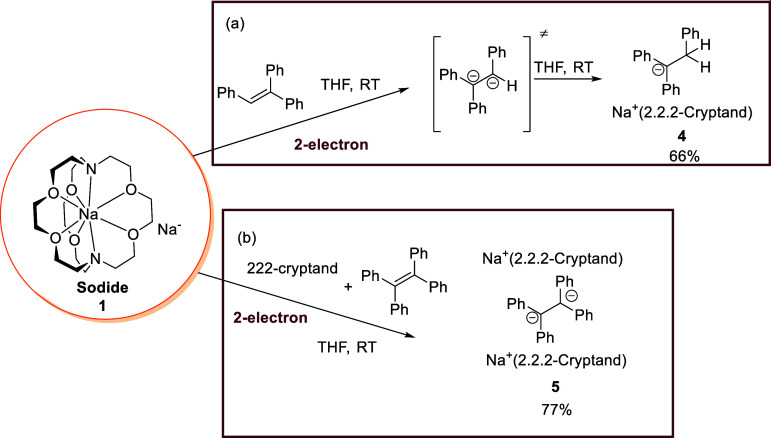
Reactions between the sodide **1** with (a) 1,1,2-triphenylethylene; (b) 1,1,2,2,-tetraphenylethylene.

**Figure 5:**
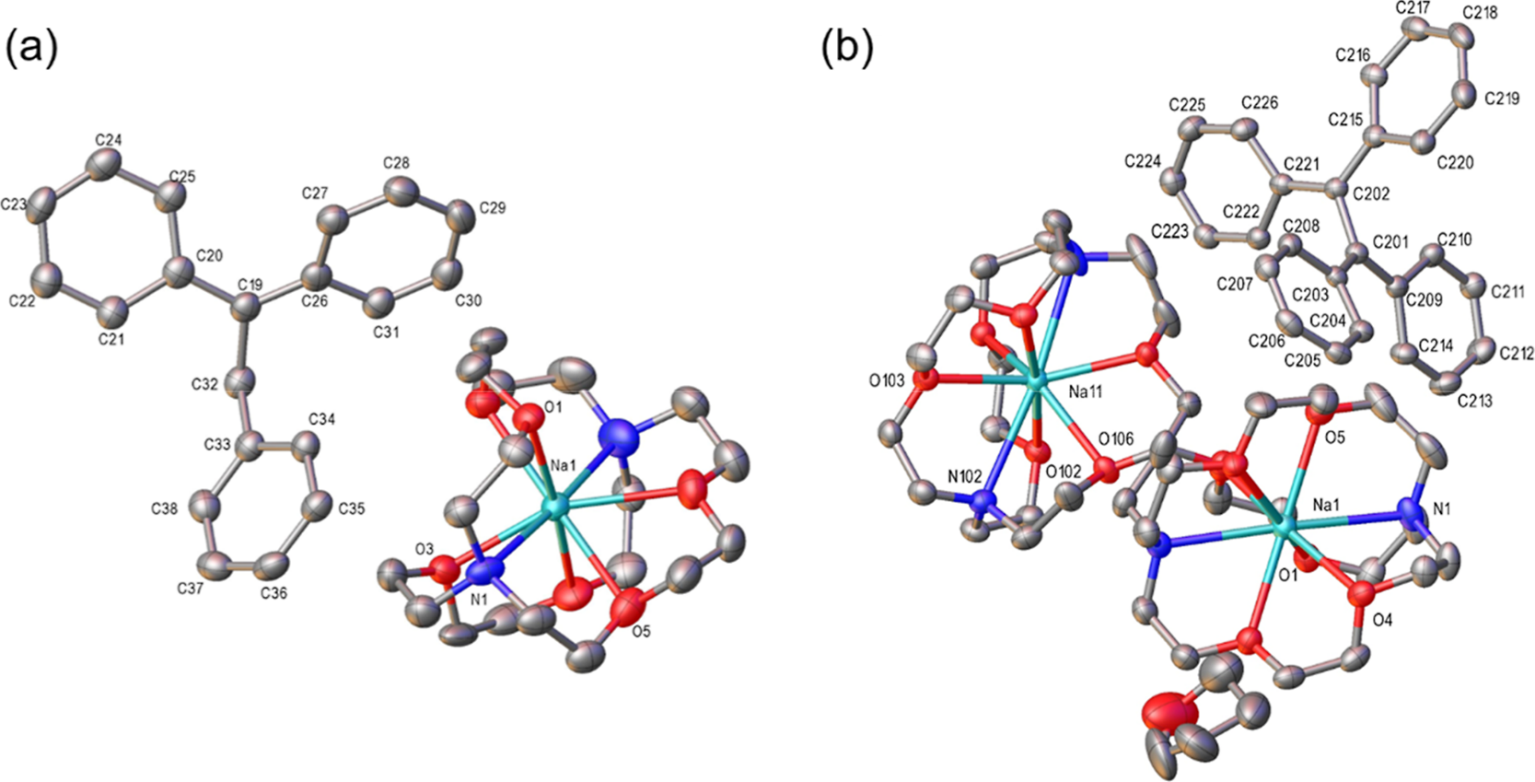
(a) Crystal structure of [Na^+^(2,2,2-cryptand)]­[Ph_2_C–CH_2_Ph]^−^ (**4)** at 50% thermal ellipsoid. Key bond lengths (Å): C(19)–C(20) 1.438(3), C(19)–C(32) 1.528(3), C(19)–C(26) 1.431(3), C(33)–C(32) 1.512(3). Key bond angles (°): C(34)–C(33)–C(32) 120.8(2), C(38)–C(33)–C(34) 118.0(2), C(38)–C(33)–C(32) 121.1(2), C(20)–C(19)–C(32) 116.5(2), C(26)–C(19)–C(20) 124.9(2), C(26)–C(19)–C(32) 118.7(2), C(21)–C(20)–C(19) 121.3(2), C(25)–C(20)–C(19) 124.3(2), C(25)–C(20)–C(21) 114.3(2), C(33)–C(32)–C(19) 115.7(2). Hydrogen atoms are omitted and only the disordered component with the highest occupancy is shown for clarity; (b) crystal structure of [Na^+^(2,2,2-cryptand)]_2_[Ph_2_C–CPh_2_]^2–^ (**5)** at 50% thermal ellipsoid. Key bond lengths (Å): C201–C202 1.512(3), C201–C203 1.418(3), C201–C209 1.441(3), C202–C215 1.430(3), C202–C221 1.441(3), C203–C204 1.447(3), C203–C208 1.435(3), C204–C205 1.381(3), C205–C206 1.387(3), C206–C207 1.397(3), C207–C208 1.378(3). Key bond angles (°): C203–C201–C202 119.39(17), C203–C201–C209 123.87(18), C209–C201–C202 116.74(17), C215–C202–C201 118.27(17), C215–C202–C221 123.98(18), C221–C202–C201 117.50(17), C201–C203–C204 125.85(18), C201–C203–C208 121.50(18), C208–C203–C204 112.51(18), C202–C215–C216 125.75­(19), C220–C215–C202 120.81(19), C220–C215–C216 113.23(19). Hydrogen atoms are omitted, and only the disordered component with the highest occupancy is shown for clarity.

In order to stabilize the two-electron-reduced 1,2-dicarbanion, we moved to tetraphenylethylene, where the extra phenyl substituent would offer both better electronic and steric protection. The reaction between **1** and 1 equiv of tetraphenylene, plus 1 additional equivalent of 2,2,2-cryptand to sequester the potentially generated Na^+^, was carried out in THF at room temperature. A rapid color change from deep blue to dark purple was observed with the formation of a large amount of a red crystalline solid. This reaction yields a 1,2-dicarbanion SIP complex [Na^+^(2,2,2-cryptand)]_2_[Ph_2_C–CPh_2_]^2–^ (**5**) in 77% yield (Figure 4b). **5**’s structure was unequivocally characterized by the SCXRD study (Figure 5b). The *C*–*C* bond length of the reduced [Ph_2_C–CPh_2_]^2–^ is 1.512(3) Å, which is in the range of a single bond^40^ and is in sharp contrast with the C=C double bond of tetraphenylethylene (1.354(2) Å^41^). The C–C bond length in **5** is similar to the C–C bond length from another Na-containing SIP [Ph_2_C–CPh_2_]^2–^ species, [Na^+^(diglyme)_2_]_2_ [Ph_2_CCPh_2_]^2–^ (1.507(3) Å),^42^ however longer than the C–C bond in a contact ion pair tetraphenylethylenedisodium diethyl ether complex (1.487 Å).^43^

The dianionic nature of the reduced tetraphenylethylene was proven using not only the charge balance from the SCXRD structure but also the calculated NPA charge distribution, where the overall charge of the [Ph_2_C–CPh_2_]^2–^ is −1.924 (Figure 6). The negative charge of the [Ph_2_C–CPh_2_]^2–^ is found to be distributed over the structural moiety, instead of localizing entirely on the *C*Ph_2_ carbons, with the *ortho*- and *para*-carbons also exhibiting a similar or increased negative charge compared to the other carbon atoms. The two counter cations are shown to have a total charge of 0.961 and 0.964, respectively, giving an overall charge of 0.001 clearly indicating that **5** is a neutral complex (Figure S31d).

**Figure 6:**
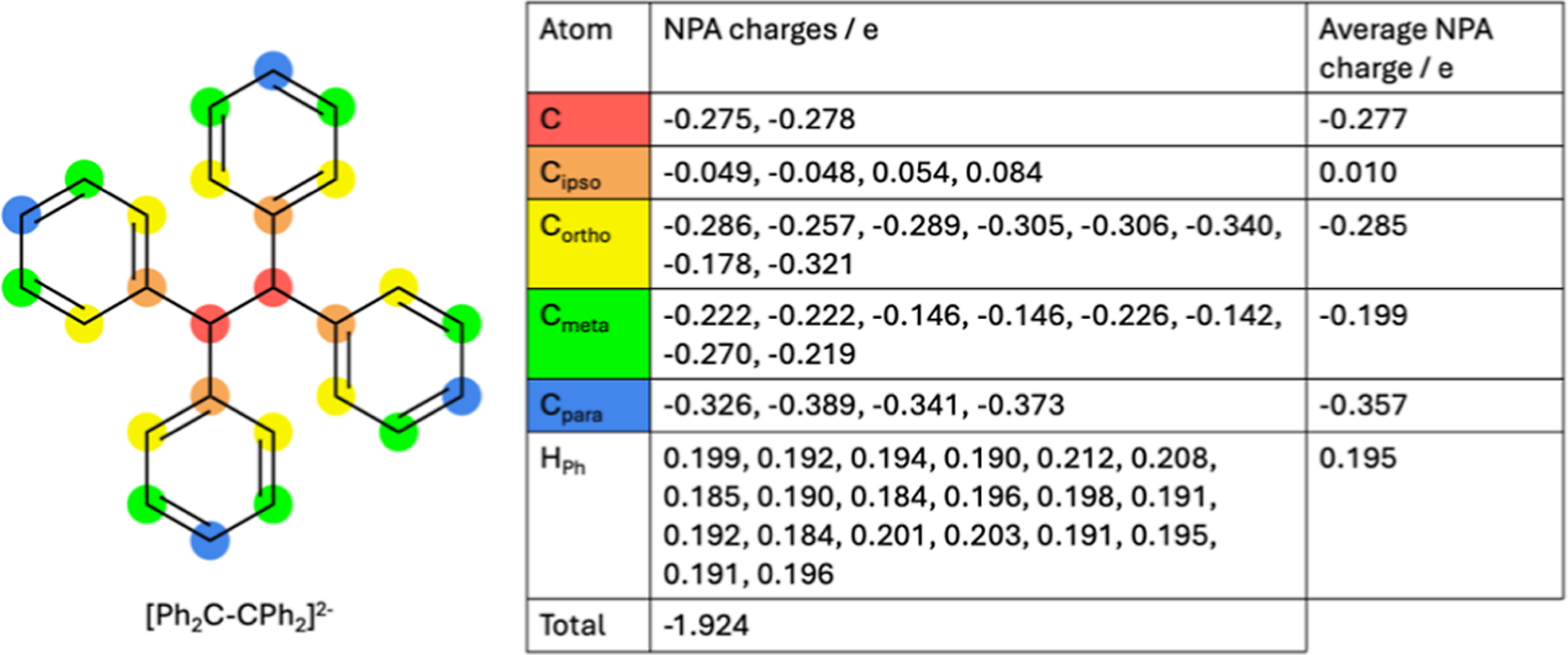
NPA charges of the [Ph_2_C–CPh_2_]^2–^ dianion.

Since the electronic structures of the olefins have a substantial influence on their reduction outcome with the sodide, it is intriguing to study the reactions between **1** and a cyclic antiaromatic conjugate multiolefin. We chose 1,3,5,7-tetracyclooctatetrene (COT) as the model substrate (Figure 7).^44^

**Figure 7:**
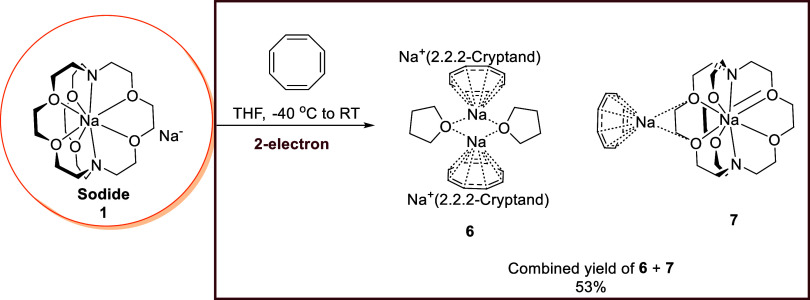
Reaction between the sodide **1** with COT.

The reaction between **1** and COT afforded a mixture of two complexes, **6** and **7**, which both crystallize out at room temperature and both of which feature a dianionic cyclooctatetraenide ligand, i.e., a 2-electron reduction occurred (Figure 7). Enabled by their different colors, single crystals of **6** (yellow) and **7** (lime green) can be manually separated under microscope and separately subjected to SCXRD. Due to their difficulty to be separated by eye and manually, a combined yield is given here, as **6** and **7** share the same structural unit [Na^+^(2,2,2-cryptand)]­[Na^+^(COT^2–^)], despite their different coordination geometries. Interestingly, upon dissolving in *d*_*8*_-THF, the mixture of **6** and **7** gives a highly symmetric ^1^H NMR spectrum, displaying only one set of signals for 2,2,2-cryptand and one single broad signal for COT^2–^ at 5.63 ppm with an approximate half-width of 0.6 ppm (Figure S20 in the Supporting Information). In addition, the ^23^Na NMR spectrum exhibits only one broad signal at −16.50 ppm, with an approximate half-width of 5 ppm (Figure S21). We postulate this is due to the presence of dynamic geometry exchange, possibly also coordination–dissociation equilibria, especially in a coordinative solvent *d*_*8*_-THF. Such equilibria are common in the NMR spectra of group-1 metal complexes.^45,46^ Our attempt to obtain variable temperature NMR spectra of the mixture of **6** and **7** was unsuccessful due to the fact that **6** and **7** crystallize out from their *d*_*8*_-THF solution at or slightly below room temperature.

Although there is significant aromatic stabilization energy, free COT^2–^ is thermodynamically unstable due to the strong Coulomb repulsion between the two additional electrons. However, the addition of two alkali metals cations can overcome this barrier and produce thermodynamically favorable complexes.^44,47^ Indeed, there are a number of alkali metal COT^2–^ and substituted COT^2–^ complexes reported in the literature, with the vast majority featuring alkali metal cations coordinated on both sides to the COT^2–^ moiety in an inverse sandwich manner.^48–54^ In contrast, in **6** and **7,** only one Na^+^ cation is directly coordinating to the COT^2–^, while the other is sequestered in a 2,2,2-cryptand ligand.

During the geometry optimization process of **6**, the two counterion [Na^+^(2,2,2-cryptand)] species migrated to above and below the two COT^2–^ species resulting in a sandwich type complex (unlike the crystal structure shown in Figure 8a), likely due to the freedom afforded the species in the gas-phase calculation when compared to the solid-state structure. Free energies of formation of both **6** and **7** were calculated (methodology outlined in Section 2.4 in the Supporting Information) suggesting that both structures are significantly stabilized relative to the starting materials, with only a small difference in the energy (5.7 kcal mol^–1^ per [Na^+^(2,2,2-cryptand)]­[Na^+^(COT^2–^)] unit), favoring **7** between the two. This small energy difference may also suggest the presence of a dynamic equilibrium between the two structures depending on the availability of THF molecules. NPA charge analysis of **6** and **7** suggests uniform delocalization of the −2 charge across all eight carbons of the COT (see Figure S31e,f). No evidence of a Na–Na bond in **6** could be observed in the calculation, suggesting all interactions are electrostatic in nature.

**Figure 8:**
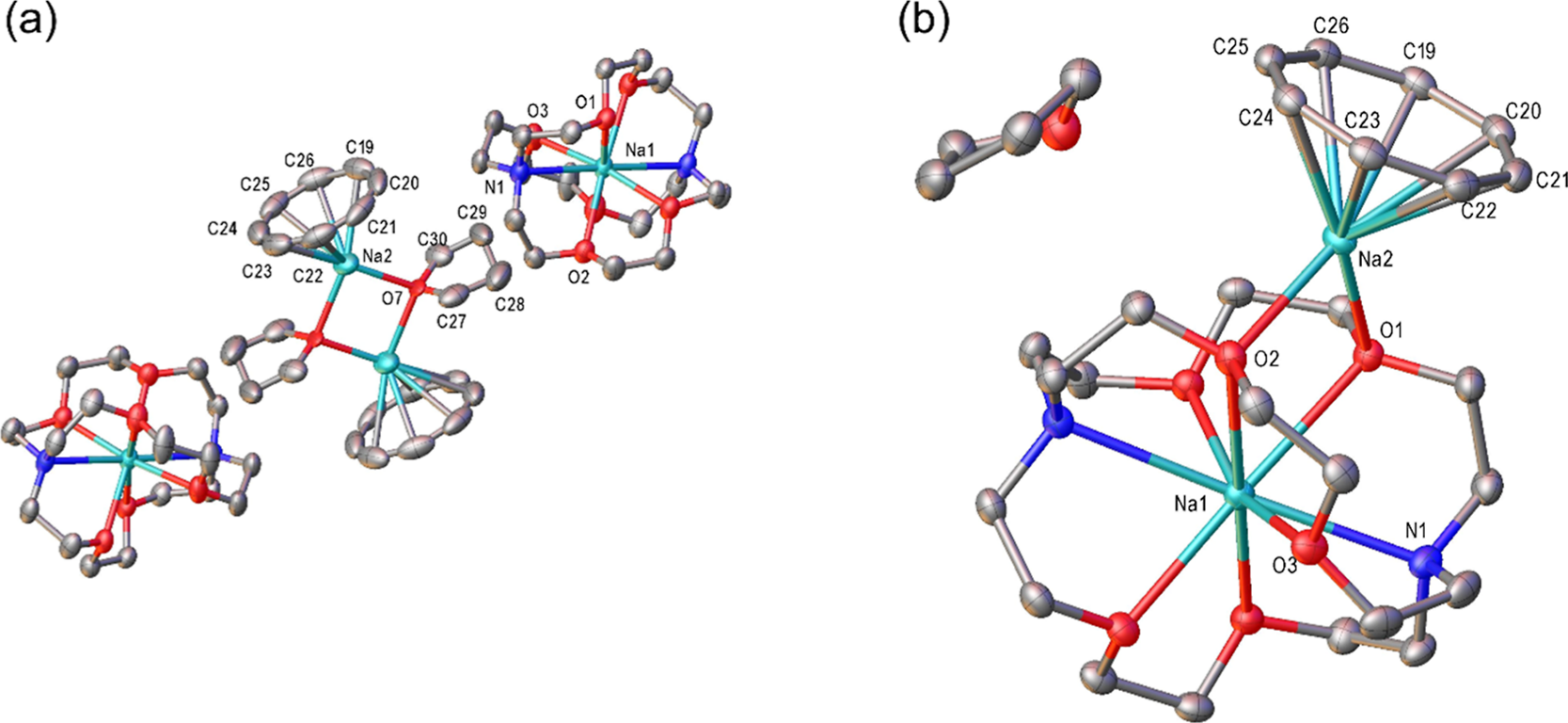
(a) Crystal structure of [Na^+^(2,2,2-cryptand)]_2_[Na^+^(COT^2–^)­(THF)]_2_ (**6**) at 50% thermal ellipsoid. Key bond lengths (Å): C19–C20 1.411(8), C19–C26 1.414(8), C19–Na2 2.686(4). Key bond angles (°): C20–C19–C26 135.29(12), C19–C20–C21 134.80(13). Hydrogen atoms are omitted and only the disordered component with the highest occupancy is shown for clarity. The Na­(C_8_H_8_)­(THF)]_2_ complex is located on an inversion center such that only half is crystallographically unique, with the THF ligand in this complex also being disordered over three positions. The structure contains one molecule of unbound THF per [Na­(C_8_H_8_)­(THF)]_2_ complex, disordered over two positions across a 2-fold rotation axis. (b) Crystal structure of [Na^+^(2,2,2-cryptand)]­[Na^+^(COT^2–^)] (**7**) at 50% thermal ellipsoid. Key bond lengths (Å): Na1–N1 2.7127(12), Na1–Na2 3.9126(7), Na1–O1 2.6341(10), Na1–O2 2.7749(10), Na1–O3 2.4109(10), Na1–O5 2.5414(10), Na2–O1 2.3572(9), Na2–O2 2.3654(10), C19–C20 1.410(2), C19–C26 1.411(2), C19–Na2 2.6602(14), C20–C21 1.411(2), C20–Na2 2.6744(14), C21–C22 1.415(2), C21–Na2 2.6233(14), C22–C23 1.412(2), C22–Na2 2.5631(14), C23–C24 1.408(2), C23–Na2 2.5764(13), C24–C25 1.4143(19), C24–Na2 2.6249(13), C25–C26 1.4154(19), C25–Na2 2.6347(14), C26–Na2 2.6388(14). Key bond angles (°): C20–C19–C26 134.5(4), C19–C20–C21 133.6(4), C22–C21–C20 134.7(4), C23–C22–C21 137.1(4), C22–C23–C24 135.6(4), C23–C24–C25 134.9(4), C26–C25–C24 135.0(4), C25–C26–C19 134.6(4). Hydrogen atoms are omitted for clarity.

## Conclusion

In conclusion, we report the reactivity study of an isolated sodium anion complex toward five C=C double bond substrates. The Na^–^ center exhibits versatility, acting as both a 1-electron and 2-electron reductant. For phenyl-substituted ethylenes, the outcomes depend on the number of phenyl substituents: while 1,1-diphenylethylene and stilbenes undergo 1-electron reduction, the more substituted tri- and tetra-phenylethylene undergo concerted 2-electron reduction. A 2-electron reduction also occurred toward 1,3,5,7-tetracyclooctatetrene. Further work is underway to expand the reactivity of the sodide toward carbon–heteroatom unsaturated bonds, as well as utilizing the sodide as a 1- or 2-electron reductant in inorganic reductions.

## Experimental Section

### General Procedures

All manipulations were carried out in a glovebox equipped with an −40 °C freezer and a cold well, under an atmosphere of dry argon. Solvents were dried with molecular sieves and kept in the glovebox. Chemicals were purchased from Merck, Fluorochem, or Alfa Aesar and dried under dynamic vacuum for several hours (for solids) or with activated 4 or 3 Å molecular sieves that had been frozen, thawed, and vacuum degassed (for liquid) prior to use. All glassware, including pipettes, vials, and ampules, was silylated by being treated with trimethylsilyl chloride (Me_3_SiCl), rinsed with water, and dried in a 150 °C oven for 12 h prior to use.

Solution-state ^1^H, ^23^Na, and ^13^C­{^1^H} NMR spectra were recorded on a Bruker AVIII console (2009) or a Bruker AVANCE NEO console (2017) spectrometer operating at 400 MHz for ^1^H NMR, 106 MHz for ^23^Na NMR, and 101 MHz for ^13^C­{^1^H} NMR.

All yields are calculated based on the organic substrates.

Continuous-wave (CW) EPR spectra were recorded on a Bruker EMX EPR spectrometer and ESR5000 benchtop operating at the X-band frequency (9.4 GHz). Field corrections were applied to all spectra using Bruker’s strong pitch (*g* = 2.0028) as a reference. EPR spectrum simulations were performed by using Easyspin software.

UV–vis absorbance spectra were recorded on a Cary 3500 multicell UV–vis spectrophotometer.

Powder X-ray diffraction (PXRD) measurements were performed on a Stoe Stadi-P diffractometer (Stoe & Cie GmbH, Darmstadt, Germany) in transmission capillary mode, using monochromated Mo Kα1 radiation (λ = 0.7093 Å). Samples were packed in Kapton capillaries (0.5 mm) and rotated during data collection. Data were recorded over a 2θ range of 1°–30° with a moving size of 6° and a total acquisition time of about 32 min.

**Caution!** Complexes **1–7** are air-sensitive and potentially pyrophoric and should be handled under an inert atmosphere. If these complexes are exposed to air, ensure no flammable materials are in the vicinity. Care should be taken both in the handling of the cryogen liquid nitrogen and its use in the in-line trap, when drying the complexes under vacuum, to avoid the condensation of oxygen from air.

[Na^+^(2,2,2-cryptand)]­Na^–^ (**1**) was prepared, as previously described.^21^

For the computational methods and spectroscopic data, please find more detailed information in the Supporting Information.

### Reaction of [Na^+^(2,2,2-Cryptand)]­Na^–^ (**1**) with 1,1-Diphenylethylene to Form [Na^+^(2,2,2-Cryptand)]_2_[Ph_2_CCH_2_CH_2_CPh_2_]^2–^ (**2**)

At room temperature, a colorless solution of 1,1-diphenylethylene (0.3 mmol, 54.0 mg in 4 mL of THF) was added into **1** (0.3 mmol, 126.7 mg) in a one-portion manner, which resulted in the formation of a red solution, from which a red crystalline solid appeared immediately. The reaction was stored at −40 °C for 24 h, resulting in red single crystals suitable for the SCXRD study. The mother liquor was removed, and the residual solid was washed with *n*-hexane (1 mL × 3) and dried under vacuum to afford **2** as a red crystalline solid (127.6 mg, 73% yield).

**2** is insoluble in *d*_*6*_-benzene, *d*_*8*_-THF, and *d*_*8*_-toluene and decomposes in CDCl_3_; hence, its NMR spectra are not available. In this case, PXRD was measured to further prove the bulk purity.

### Reaction of [Na^+^(2,2,2-Cryptand)]­Na^–^ (**1**) with *trans*-Stilbene and *cis*-Stilbene to Form [Na^+^(2,2,2-Cryptand)]­[PhCH–CHPh]^•–^ (**3**)

At room temperature, a colorless solution of *trans*-stilbene (0.1 mmol, 18.0 mg in 4 mL of THF) was added into **1** (0.1 mmol, 42.2 mg) with stirring to afford a deep brown solution. The solution was stirred at room temperature for 24 h. The mixture was filtered, and 2 mL of *n*-hexane was added to the solution, which was kept still at room temperature for 6 days, to afford a green crystalline solid. The mother liquor was removed, and the residual solid was washed with *n*-hexane (1 mL × 3) and dried under vacuum, to afford **3** as a green crystalline solid (83.4 mg, 72%).

Single crystals suitable for the SCXRD study were obtained from a separated reaction (*trans*-stilbene 0.1 mmol, 18.0 mg in 3 mL of THF; **1** 0.1 mmol, 42.2 mg). The crude product was crystallized in a mixture of THF: *n*-hexane (4 mL: 2 mL) at room temperature.

At room temperature, a colorless solution of *cis*-stilbene (0.1 mmol, 18.0 mg in 4 mL of THF) was added into **1** (0.1 mmol, 42.2 mg) with stirring to afford a deep brown solution. The solution was stirred at room temperature for 24 h. The reaction mixture was filtered, and 2 mL of *n*-hexane was added to the obtained solution, which was kept still at room temperature for 6 days, to afford green single crystals suitable for the SCXRD study. The mother liquor was removed, the residual solid was washed with *n*-hexane (1 mL × 3) and dried under vacuum, to afford **3** as a green crystalline solid (35.8 mg, 62%). The **3** was a paramagnetic, NMR silent species. Due to the paramagnetic nature of **3**, the NMR spectra showed only large solvent peaks and broad peaks of 222-cryptand.

^1^H NMR (*d*_*8*_-THF, 25 °C, 400.07 MHz): δ (ppm) 3.41–3.32 (m, broad, 24H, Cryptand), 2.54 (s, broad, 12H, Cryptand).

^23^Na NMR (*d*_*8*_-THF, 25 °C, 115.83 MHz): δ (ppm) −9.39.

### Reaction of [Na^+^(2,2,2-Cryptand)]­Na^–^ (**1**) with Triphenylethylene to Form [Na^+^(2,2,2-Cryptand)]­[Ph_2_C–CH_2_Ph]^−^ (**4**)

At room temperature, a colorless solution of triphenylethylene (0.1 mmol, 25.6 mg in 4 mL of THF) was added into **1** (0.1 mmol, 42.2 mg), which was stirred at room temperature 24 h. A dark reddish-purple solution was obtained with a small amount of the solid. The reaction mixture was filtrated through a glass wool Celite pad, followed by the addition of 2 mL of *n*-hexane. The solution was stored at −40 °C for 6 days to afford black crystals which were suitable for the SCXRD study. The mother liquor was removed, and the crystals were washed with Et_2_O (2 mL × 3) at room temperature and then dried under vacuum, to afford **4** as a greenish black crystal (43.0 mg, 66%). The **4** was partially dissolved in *d*_8_-THF. Due to the relatively poor solubility of **4** in *d*_8_-THF, the NMR spectra showed large solvent peaks.

^1^H NMR (*d*_*8*_-THF, 25 °C, 400.07 MHz): δ (ppm) 7.26–7.00 (m, 6H, Ar*H*), 6.91–6.89 (m, 4H, Ar*H*), 6.45 (m, 3H, Ar*H*), 5.61 (m, 2H, *para*-*H*s of Ph_2_C^–^),^55^ 3.70 (s, 2H, C*H*_*2*_Ph), 3.56–3.46 (m, 24H, C*H*_*2*_ of 2,2,2-cryptand), 2.57–2.54 (m, 12H, C*H*_*2*_ of 2,2,2-cryptand).

^23^Na NMR (*d*_*8*_-THF, 25 °C, 105.83 MHz): δ (ppm) −11.03.

^13^C­{^1^H} NMR (*d*_*8*_-THF, 25 °C, 100.62 MHz): δ (ppm) 148.5, 147.3, 129.8, 128.9, 128.8, 128.6, 128.0, 127.9, 126.7, 124.4, 116.8, 107.3 (Ar*C*s), 81.5 (Ph_2_ *C* ^–^), 69.4, 68.6, 53.9 (*C*H_2_s of 2,2,2-cryptand), 39.9 (*C*H_2_Ph).

### Reaction of [Na^+^(2,2,2-Cryptand)]­Na^–^ (**1**) with Tetraphenylethylene to Form [Na^+^(2,2,2-Cryptand)]_2_[Ph_2_C–CPh_2_]^2–^ (**5**)

At room temperature, a colorless solution of the mixture of tetraphenylethylene (0.1 mmol, 32.2 mg) and 2,2,2-cryptand (0.1 mmol, 37.6 mg) in 4 mL of THF was added into **1** (0.1 mmol, 42.2 mg) with stirring. A significant amount of a red solid appeared immediately after mixing, and the mixture was stirred for 24 h at room temperature. The mixture was kept at −40 °C overnight. The mother liquor was removed, and the residual solid was washed with *n*-hexane at room temperature (2 mL × 3) and dried under vacuum to afford **5** as a red crystalline solid (86.2 mg, 77% yield), which contains single crystals suitable for the SCXRD study.

**5** is insoluble in *d*_*6*_-benzene, *d*_*8*_-THF, and *d*_*8*_-toluene, and decomposes in CDCl_3_; hence, its NMR spectra are not available.

### Reaction of [Na^+^(2,2,2-Cryptand)]­Na^–^ (**1**) with 1,3,5,7-Cyclooctatetraene to Form [Na^+^(2,2,2-Cryptand)]_2_[Na^+^(COT^2–^)­(THF)]_2_ (**6**) and [Na^+^(2,2,2-Cryptand)]­[Na^+^(COT^2–^)] (**7**)

1,3,5,7-Cyclooctatetraene (26.0 mg, 0.25 mmol) was dissolved in THF (1 mL) and cooled in an −40 °C freezer. **1** (105.6 mg, 0.25 mmol) was dissolved in THF (2 mL). The COT solution was taken out of the freezer, and the dark blue solution of **1** was added in a one-portion manner to the pale-yellow solution of COT. Immediately a slightly cloudy burgundy solution resulted. The solution was kept still at room temperature and turned green over 3 days. After 7 days at room temperature, a mixture of yellow and lime-green crystals resulted. The mother liquor was removed, and the crystals were washed with *n*-hexane (2 mL) and dried under a vacuum. A pale green crystalline solid was obtained as a mixture of **6** and **7**. Complexes **6** and **7** share the structural subunit [Na^+^(2,2,2-cryptand)]­[Na^+^(COT^2–^)] and are likely to undergo dynamic exchange in solution. Hence, the combined yield (69.5 mg, 53%) is calculated based on the subunit.

Crystals for SCXRD resulted from a reaction performed as above at 0.24 mmol scale in a total of 3 mL of THF. After 7 days at room temperature, a mixture of yellow (compound **6**) and lime-green (compound **7**) crystals resulted in the bottom of the vial; the mother liquor was removed, and both were subjected to the SCXRD study.

Note: due to the dark green color of the mother liquor, all crystals appear green by eye; however, under a microscope, a mixture of yellow and lime-green crystals was observed. While this allowed separation of the crystals for SCXRD, it was not possible to separate the mixture inside the glovebox for separate yield calculations.

^1^H NMR (*d*_*8*_-THF, 25 °C, 400.07 MHz): δ (ppm) 5.63 (s, broad, 8H, COT^2–^), 3.48–3.27 (m, 24H, C*H*_2_s of 2,2,2-cryptand), 2.49–2.34 (m, 12H, C*H*_2_s of 2,2,2-cryptand).

^23^Na NMR (*d*_*8*_-THF, 25 °C, 105.83 MHz): δ (ppm) −16.50 (br).

### Attempted Reaction of [Na^+^(2,2,2-Cryptand)]­Na^–^ (**1**) with Styrene

At room temperature, a colorless solution of styrene (0.04 mmol, 4.2 mg) in 0.5 mL of *d*_*8*_-THF was added into **1** (0.04 mmol, 16.9 mg) in one-go. The solution turned to deep blue immediately with small amounts of a deep colored solid. The reaction mixture was transferred to a J Young type NMR tube for further monitoring.

## Supplementary Material



## Notes

Reaction between **1** and 1,1-dipheneylethylene, NMR spectra, PXRD data, room temperature X-band EPR spectrum, EPR spectra, UV–vis spectra, and crystallographic data and calculation data (PDF)
