# Supplementary material for: Reactivity of a Sodium Anion with Alkenes: 1‑Electron vs 2‑Electron Reductions
Source: Inorg Chem. 2025 Dec 12;64(51):25195–204. doi: 10.1021/acs.inorgchem.5c04279 (PMC12754795; doi:10.1021/acs.inorgchem.5c04279)
Supplement: Supplementary file 1 [file ic5c04279_si_001.pdf]

Supplementary Materials for

Reactivity of a sodium anion with alkenes: 1-electron *vs* 2-electron reductions

Huanxin Zhang,[1] Nathan Davison,*[1] Jack M. Hemingway,*[2] Louise Male,[1] Paul G. Waddell,[2] Joshua Deakin,[1] Floriana Tuna,[3] James A. Dawson,[2] Erli Lu*[1]

Affiliation:

1 School of Chemistry, University of Birmingham, Edgbaston, Birmingham B15 2TT, U. K.

2 Chemistry–School of Natural and Environmental Sciences, Newcastle University, Newcastle upon Tyne NE1 7RU, U. K.

3 Department of Chemistry and Photon Science Institute, University of Manchester, Oxford Road, Manchester M13 9PL, U. K.

*Corresponding authors: jack.hemingway@newcastle.ac.uk (J. M. H.), n.davison@bham.ac.uk (N. D.), e.lu@bham.ac.uk (E. L.)

This PDF file includes:

Materials and Methods
Supplementary Text
Figures S1 to S31
Tables S1 and S2

Section 1. Experimental methods and data---S2
Section 2. Computational details and data---S20
References---S33

Materials and Methods

Section 1. extra data

1.1 Reactions of sodide

1.1.1 Reaction of sodide with 1,1-diphenylethylene

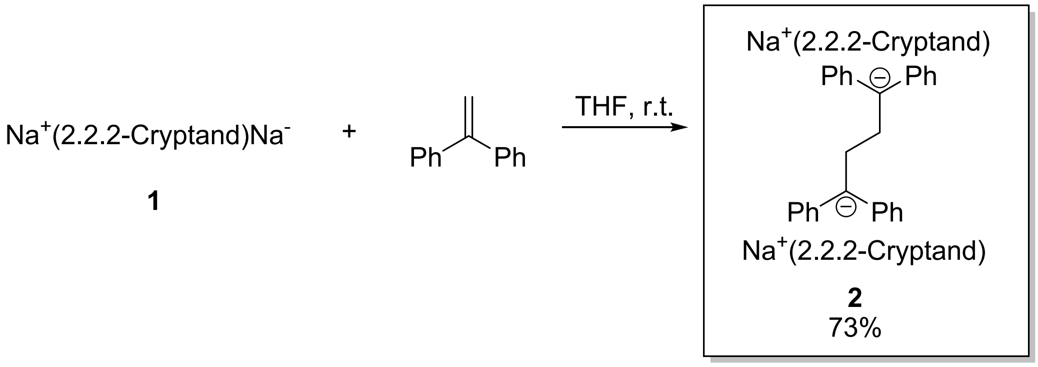

Figure S1. Reaction between **1** and 1,1-dipheneylethylene.

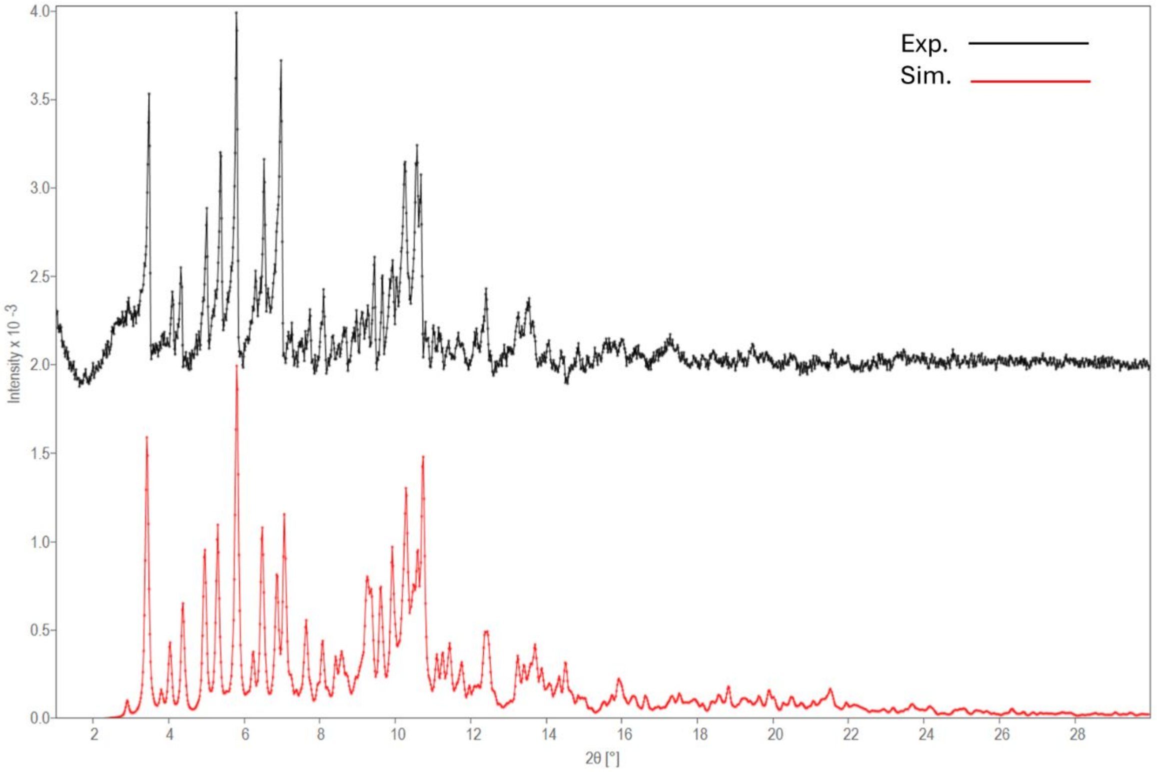

Figure S2. PXRD pattern of **2** (in black) and simulated pattern from SCXRD (in red).

1.1.2 Reaction of sodide with *trans*-stilbene and *cis*-stilbene

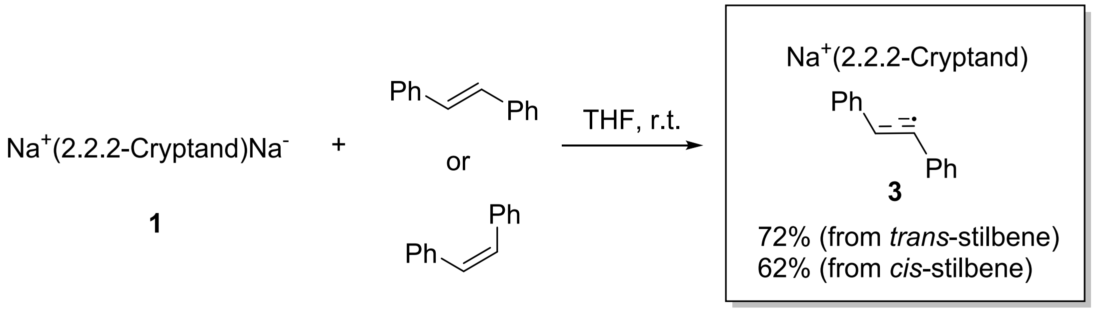

Figure S3. Reaction between **1** and *cis*- or *trans*-stilbenes.

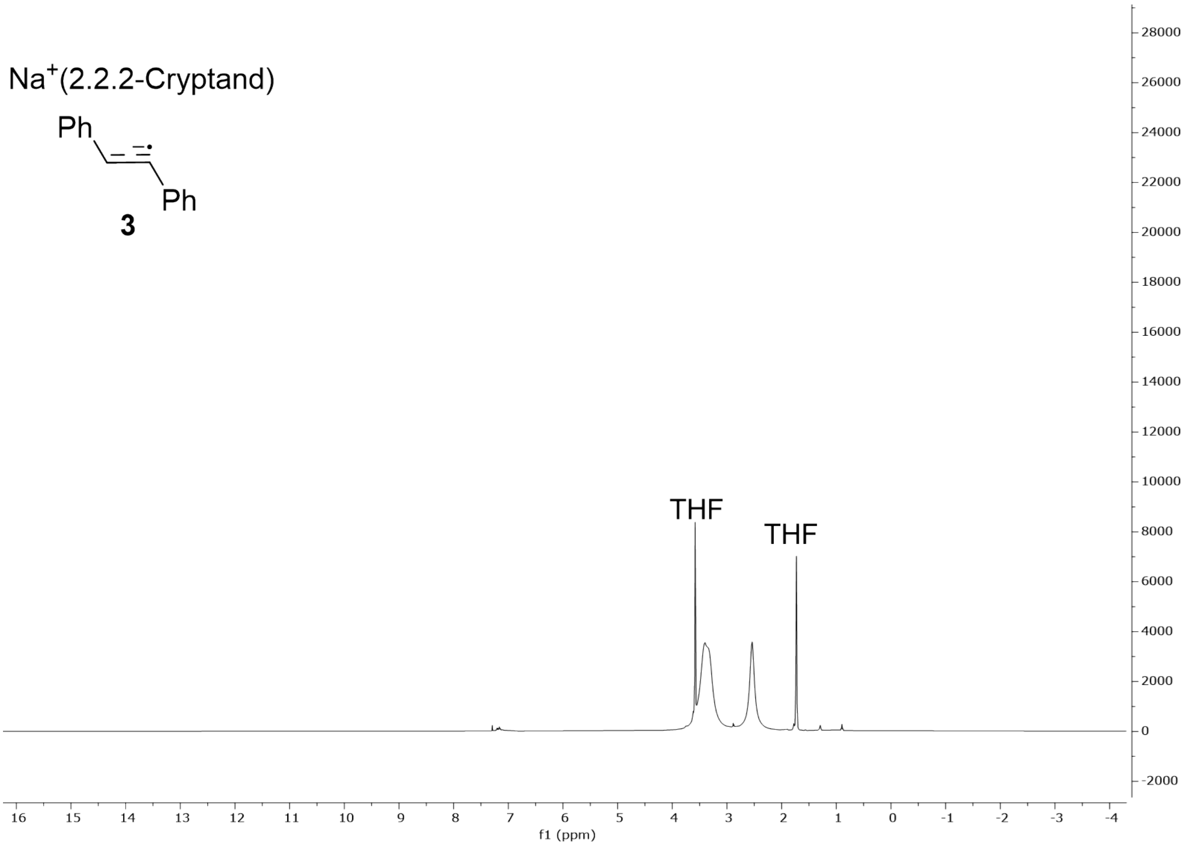

Figure S4. ^{1}H NMR (400 MHz, 25 °C) of **3** in d_8-THF.

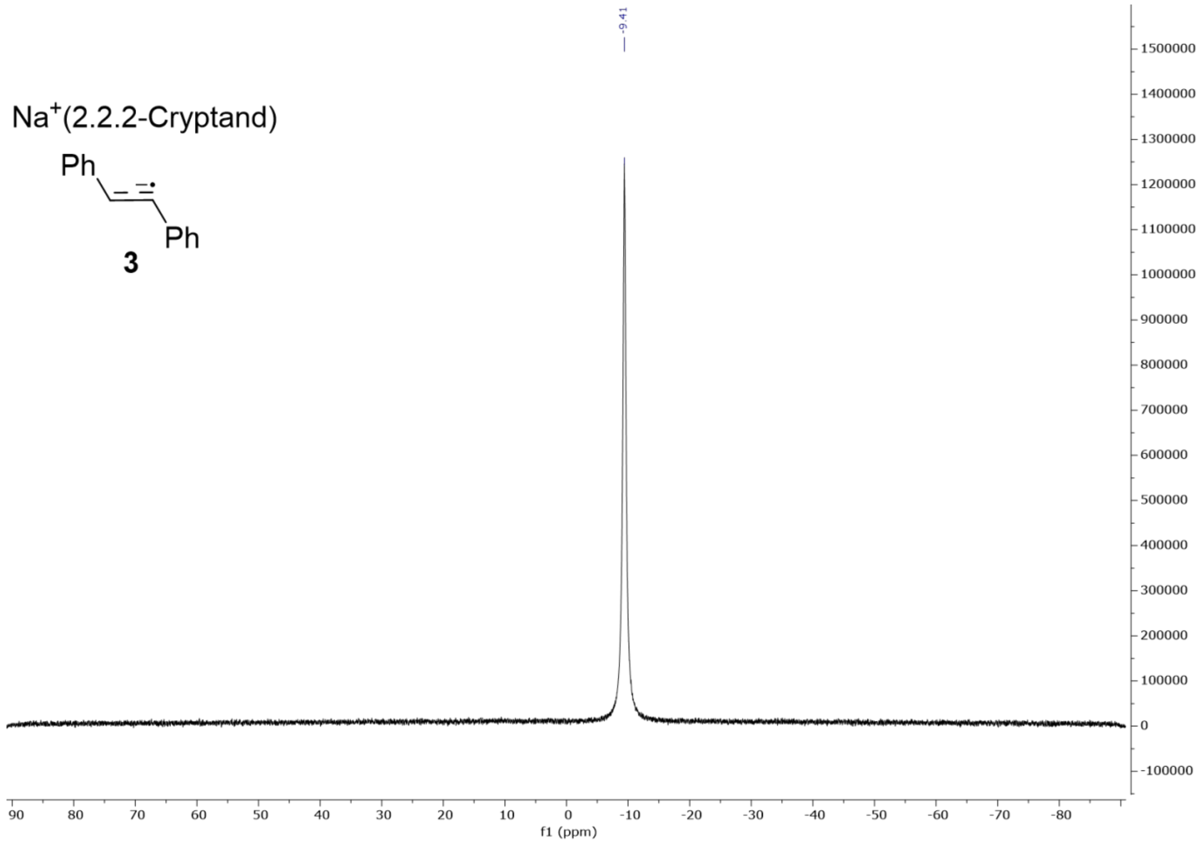

Figure S5. ^{23}Na NMR (105.83 MHz, 25 °C) of **3** in d_8-THF.

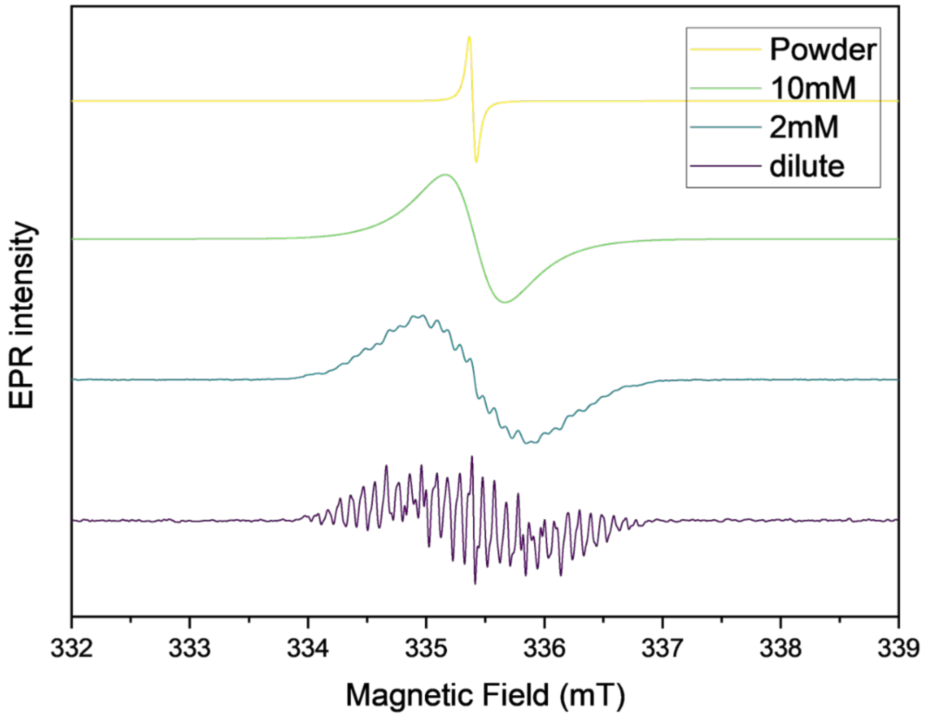

Figure S6. Room temperature X-band EPR spectrum of **3** (diluted, 2 mM, 10 mM THF solution and powder).

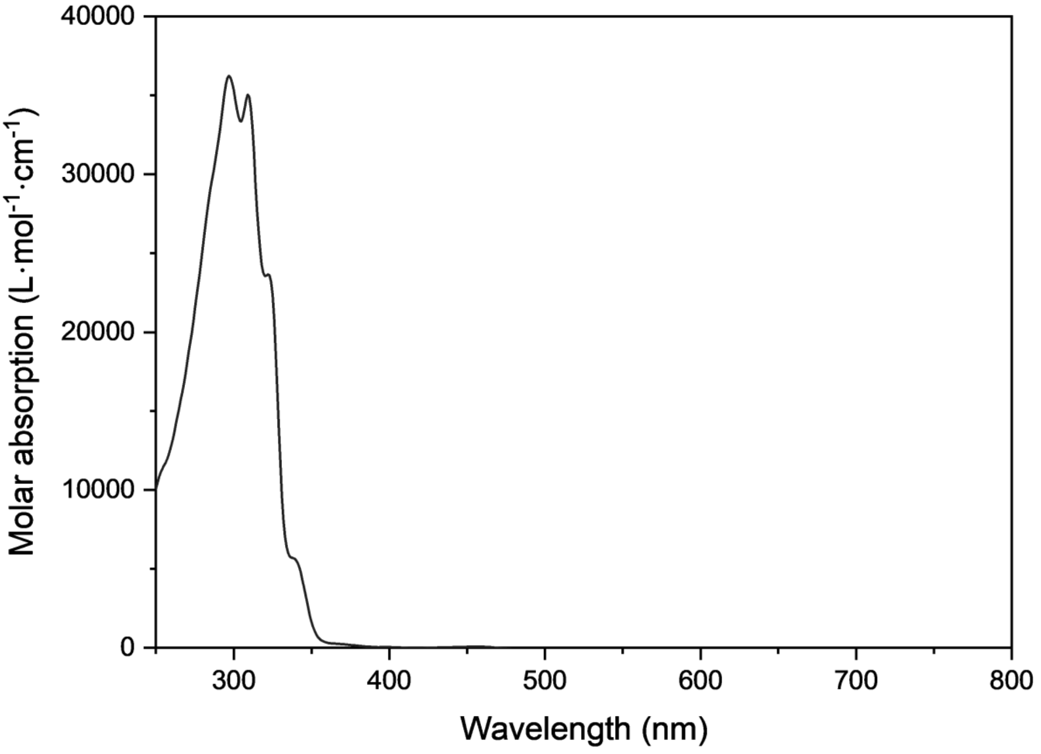

Figure S7. UV/Vis spectrum of **3** (0.04 mM THF solution, 298 K).

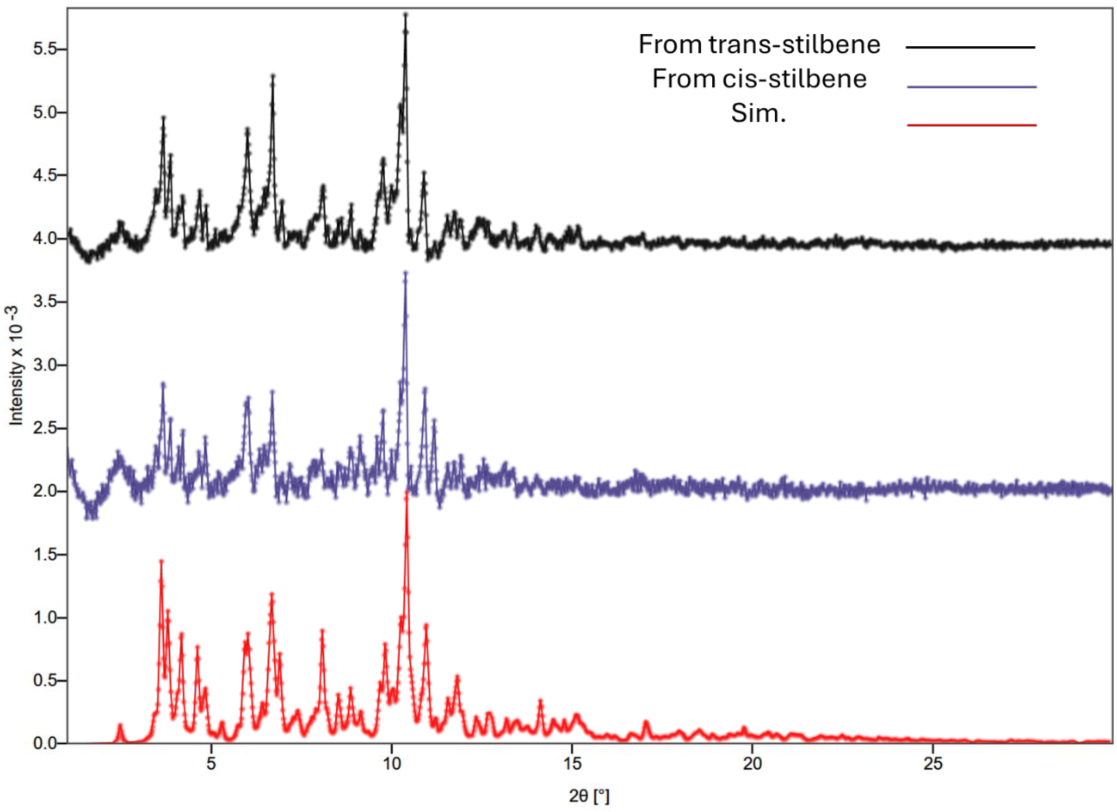

Figure S8. PXRD patterns of **3** from trans-stilbene reaction (in black), cis-stilbene reaction (in purple) and simulated pattern from SCXRD (in red).

1.1.3 Reaction of sodide with triphenylethylene

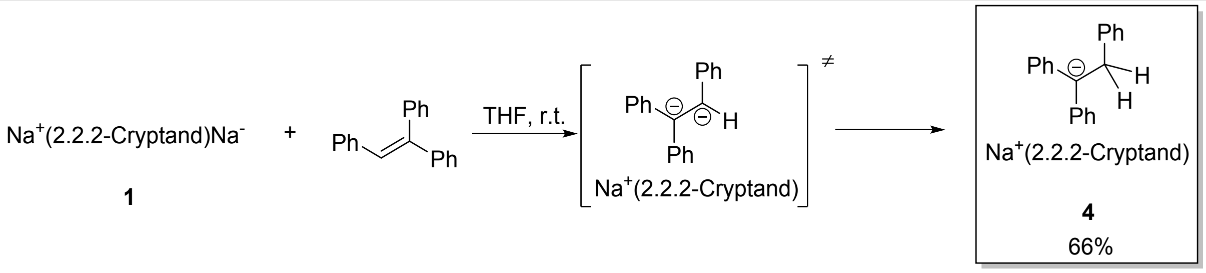

Figure S9. Reaction between **1** and 1,1,2-triphenylethylene.

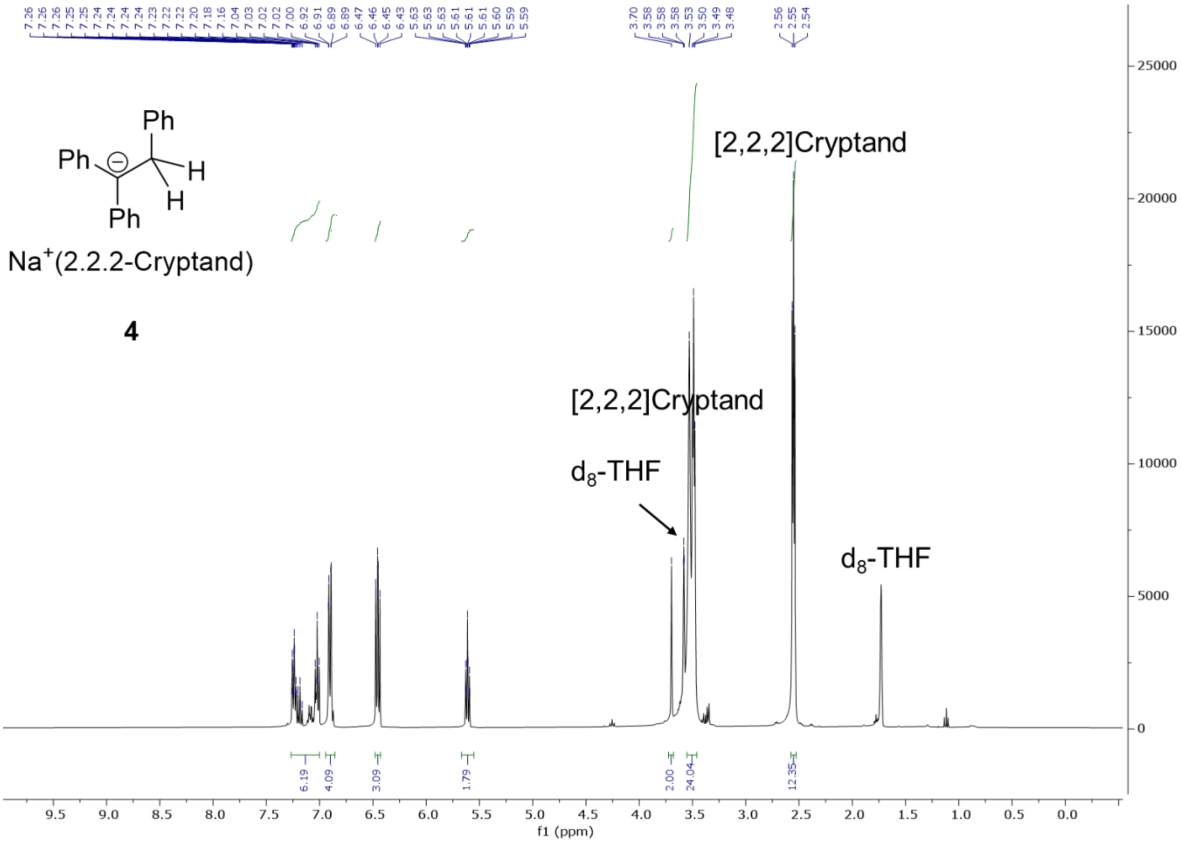

Figure S10. ^{1}H NMR (400 MHz, 25 °C) of **4** in d_8-THF.

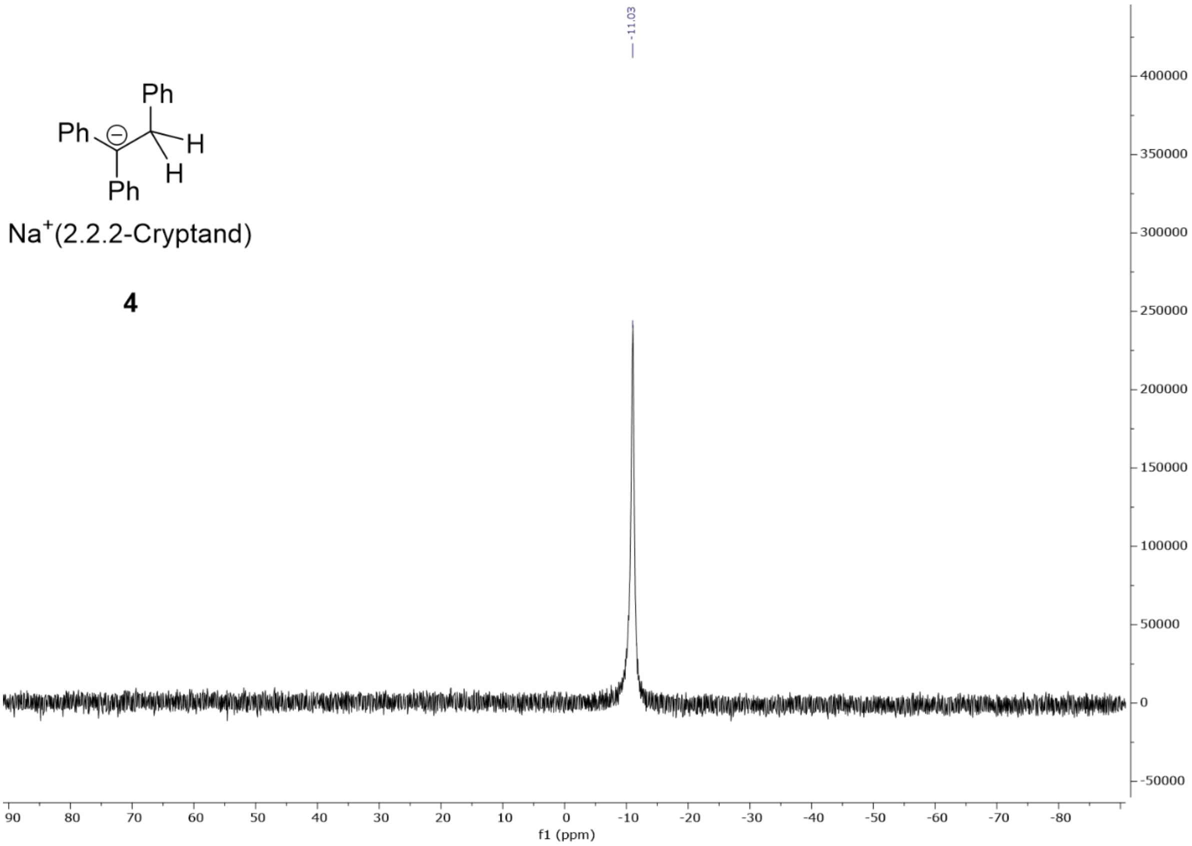

Figure S11. ^{23}Na NMR (106 MHz, 25 °C) of **4** in d_8-THF.

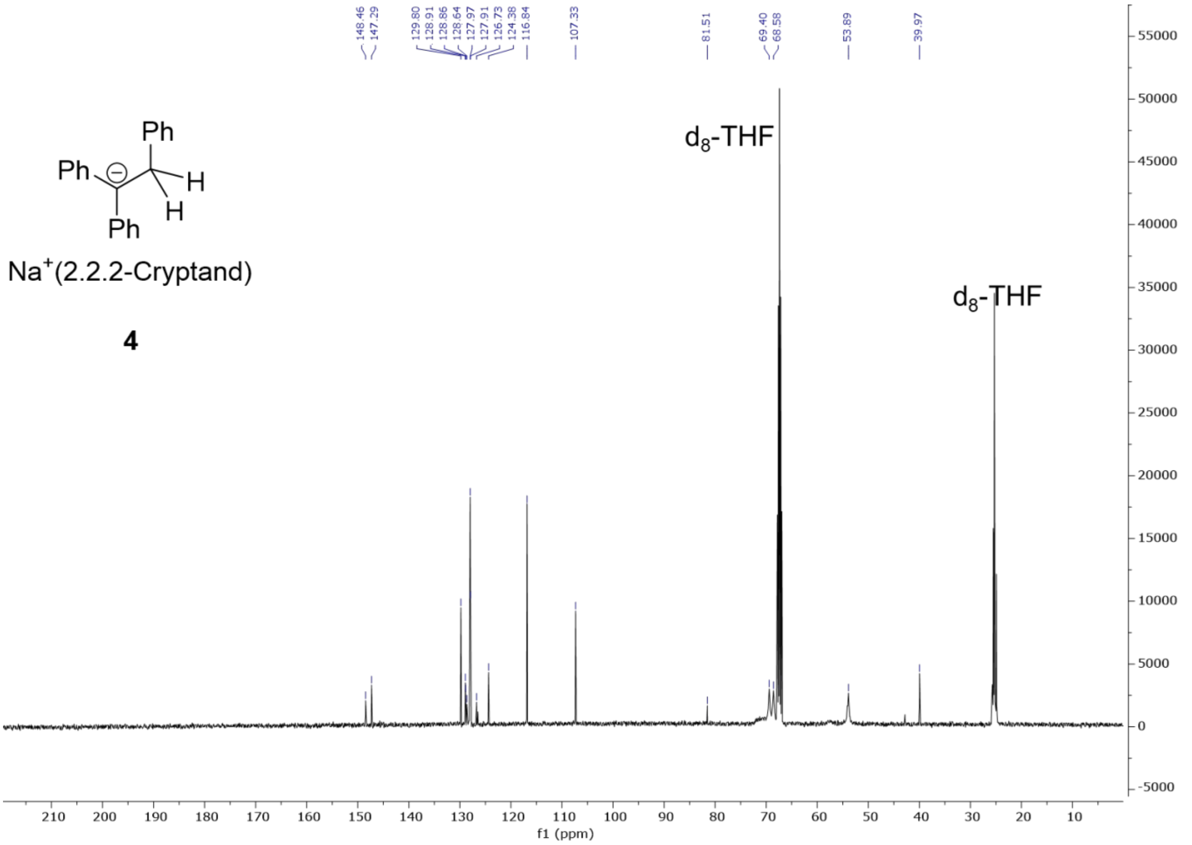

Figure S12. ^{13}C{^{1}H} NMR (101 MHz, 25 °C) of **4** in d_8-THF.

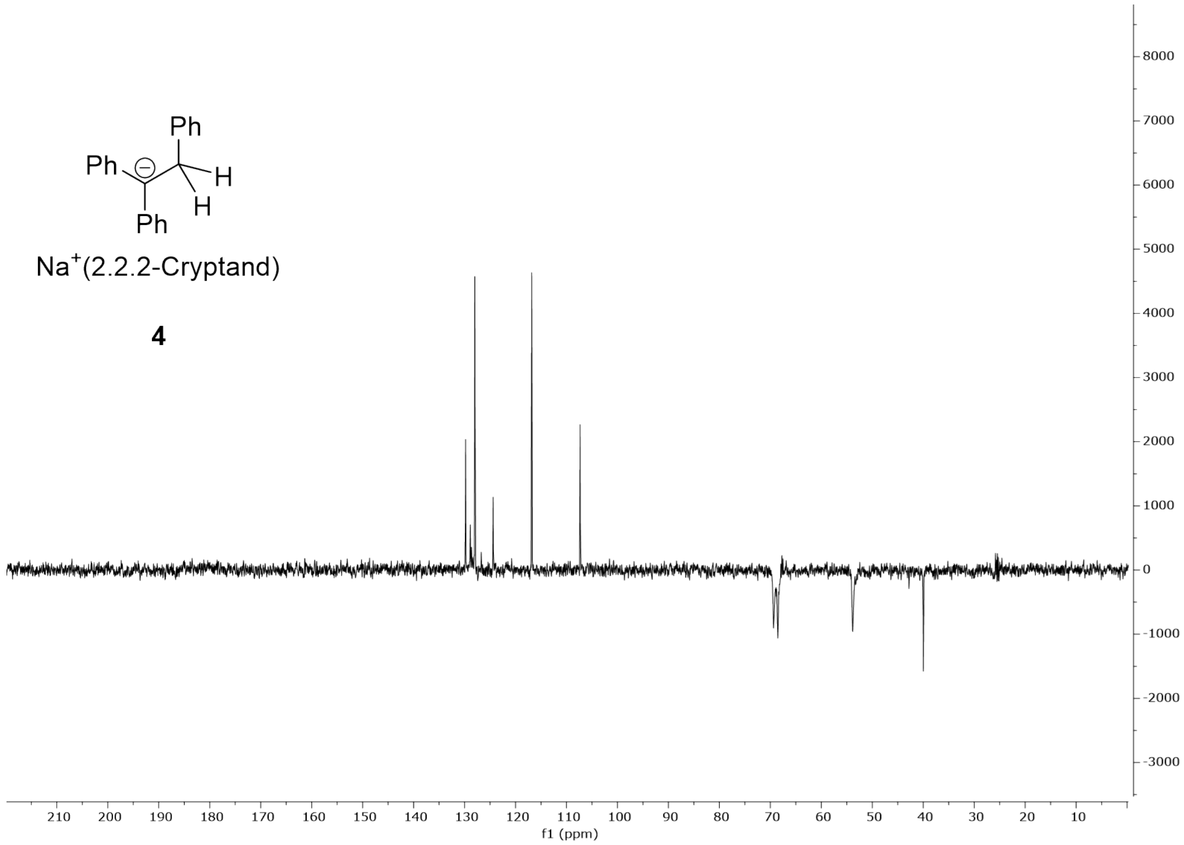

Figure S13. DEPT 135° NMR (101 MHz, 25 °C) of **4** in d_8-THF.

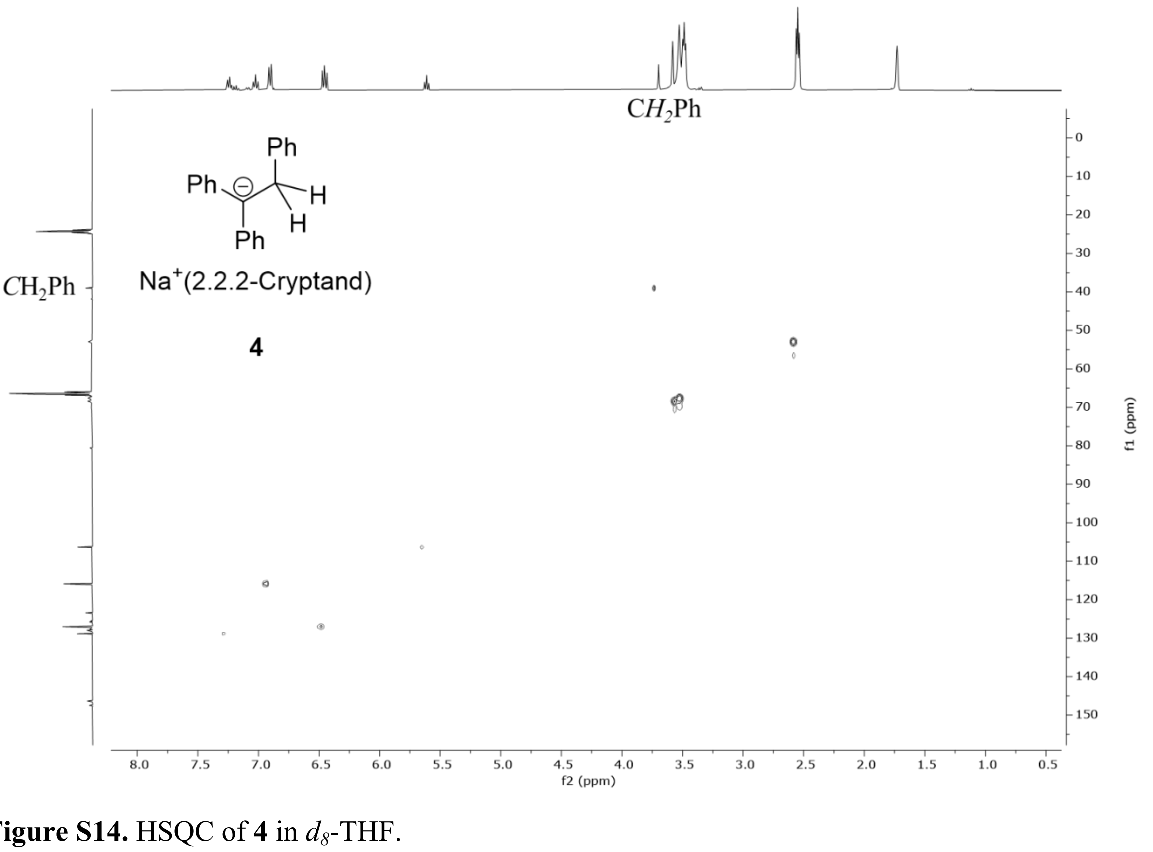

Figure S14. HSQC of **4** in d_8-THF.

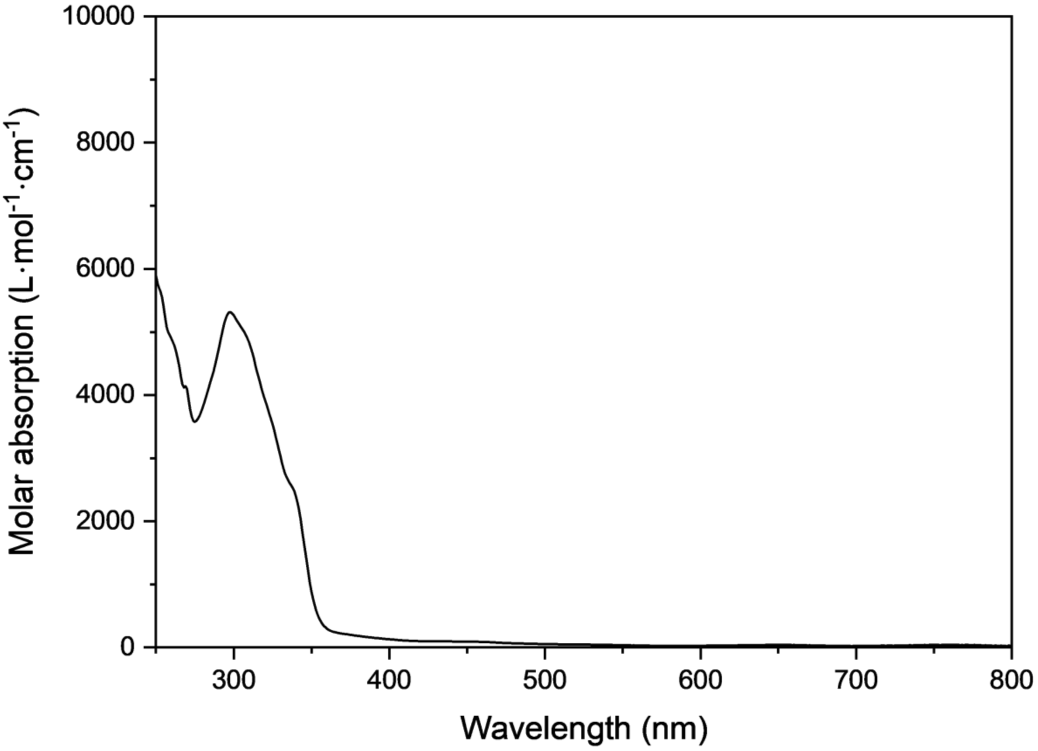

Figure S15. UV/Vis spectrum of **4** (0.2 mM in THF, 298 K).

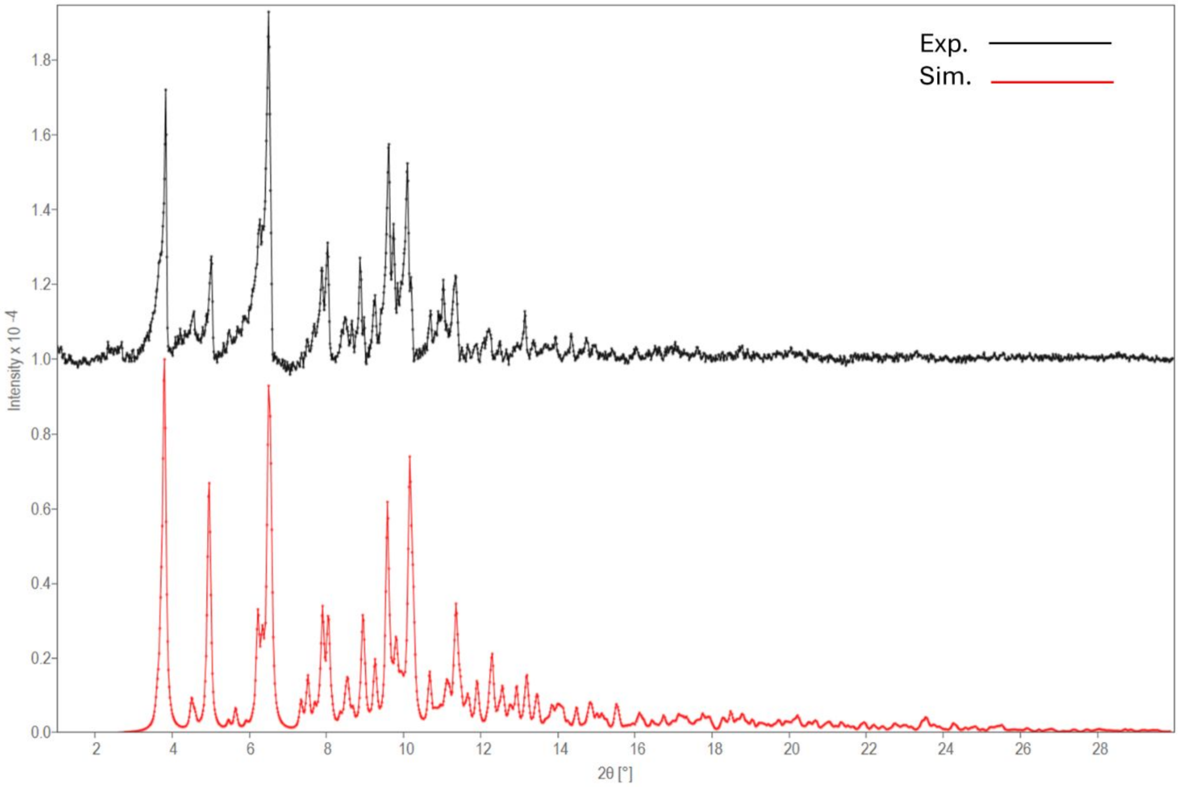

Figure S16. PXRD pattern of **4** (in black) and simulated pattern from SCXRD (in red).

1.1.4 Reaction of sodide with tetraphenylethylene

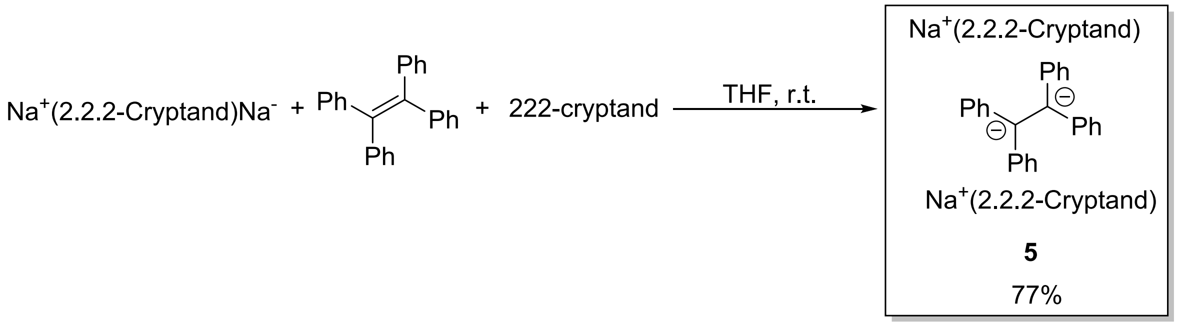

Figure S17. Reaction between **1** and 1,1,2,2-tetraphenylethylene.

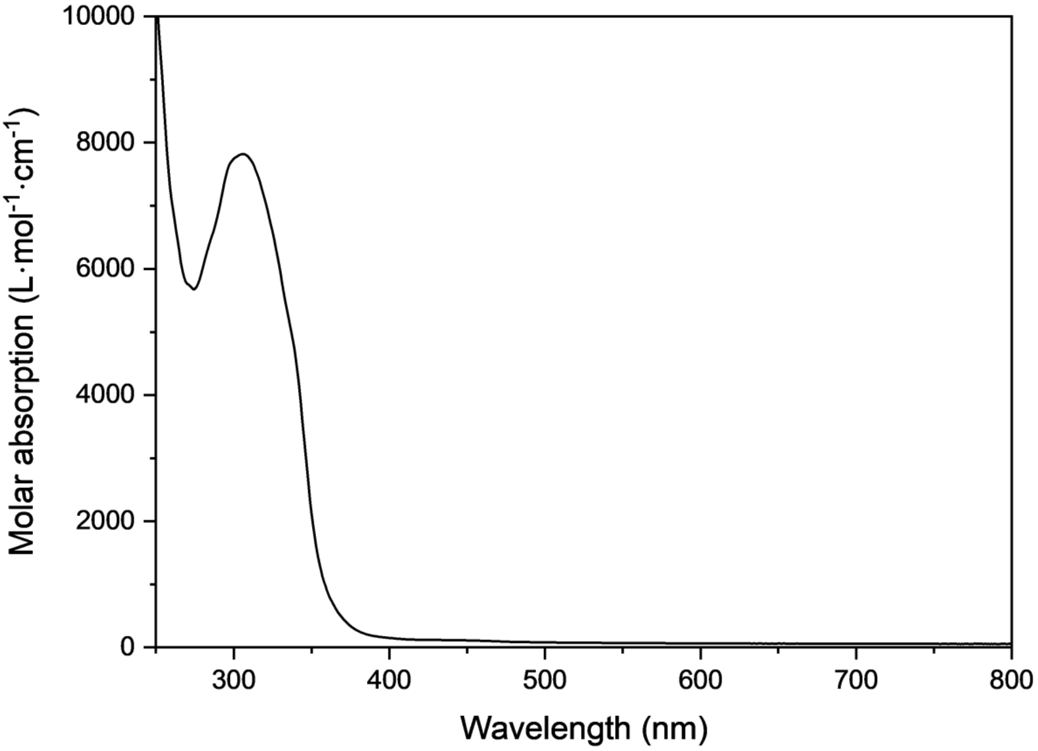

Figure S18. UV/Vis spectrum of 5 (0.2 mM in THF, 298 K).

1.1.5 Reaction of sodide with 1,3,5,7-Cyclooctatetraene

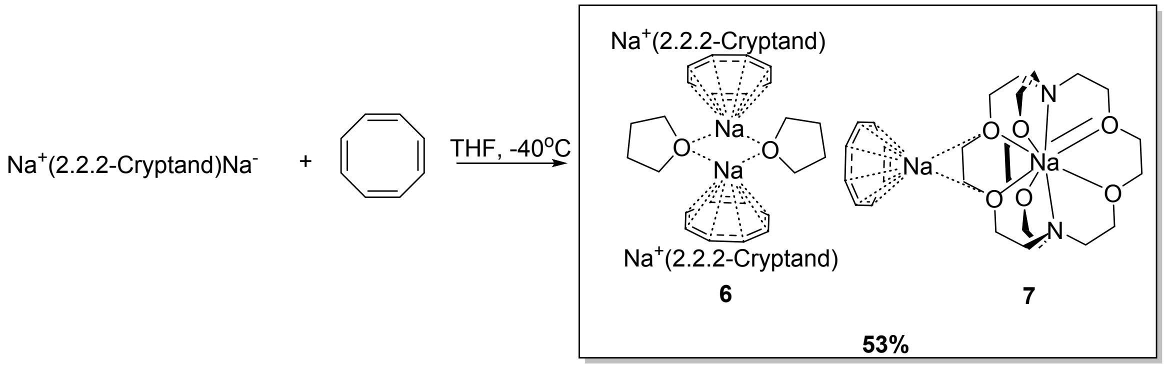

Figure S19. Reaction between **1** and 1,3,5,7-Cyclooctatetraene.

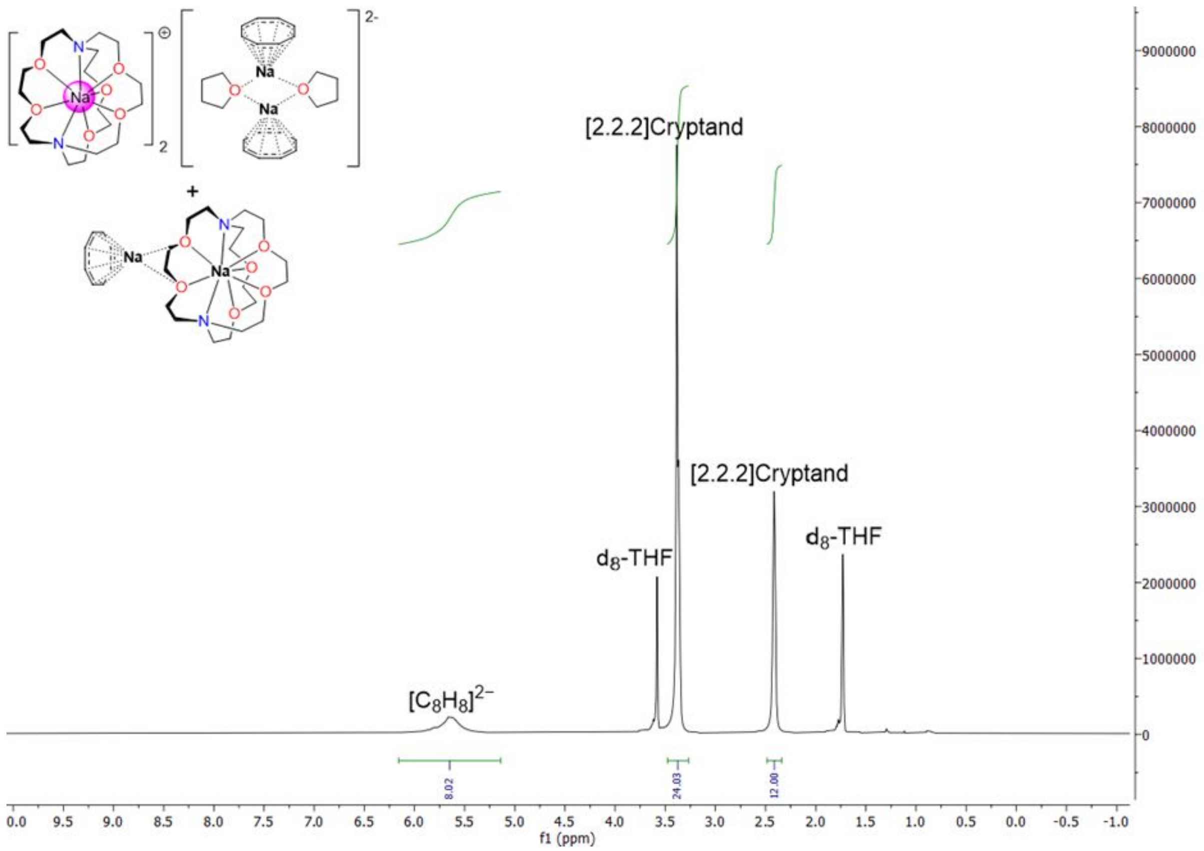

Figure S20. ^{1}H NMR (400 MHz, 25 °C) of a mixture of **6** and **7** in d_8-THF.

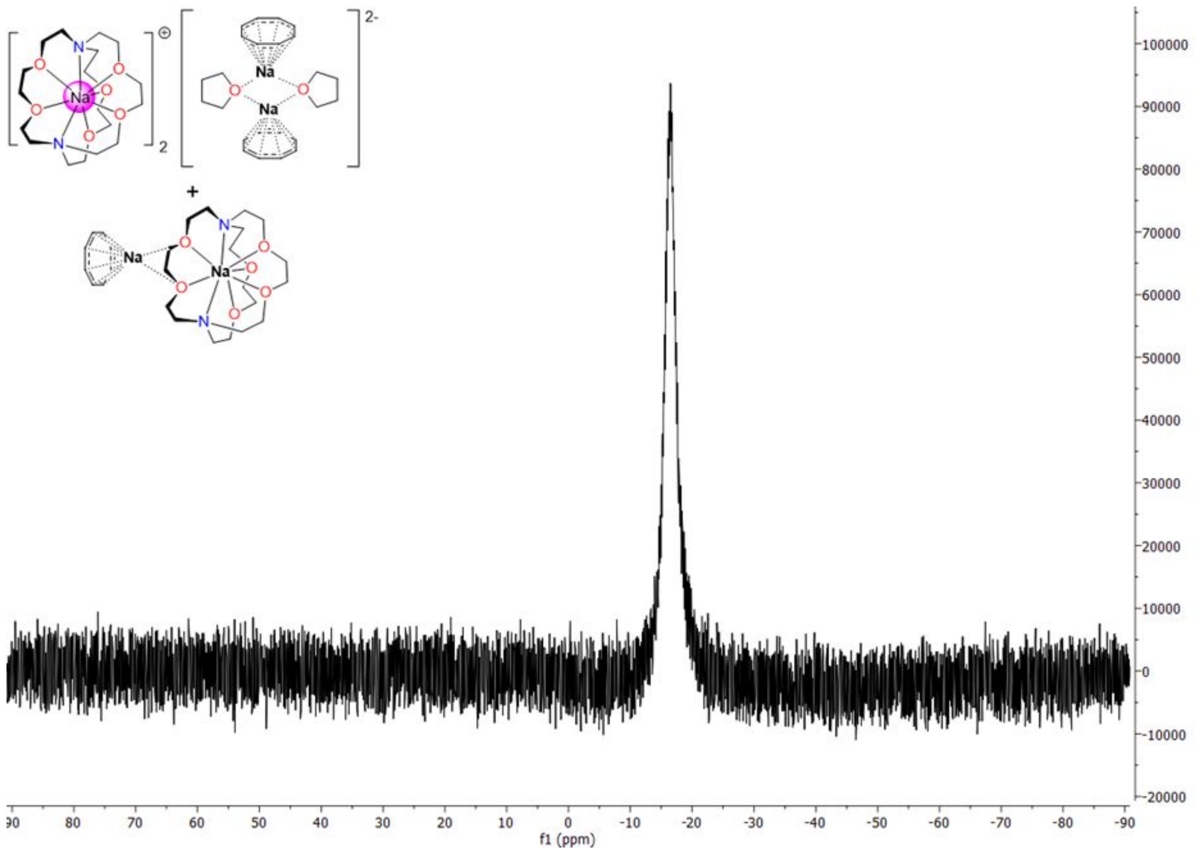

Figure S21. ^{23}Na NMR (106 MHz, 25 °C) of a mixture of **6** and **7** in d_8-THF.

1.1.6 Attempted reaction of sodide with styrene

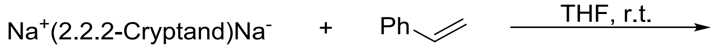

Figure S22. Attempted reaction between **1** and styrene.

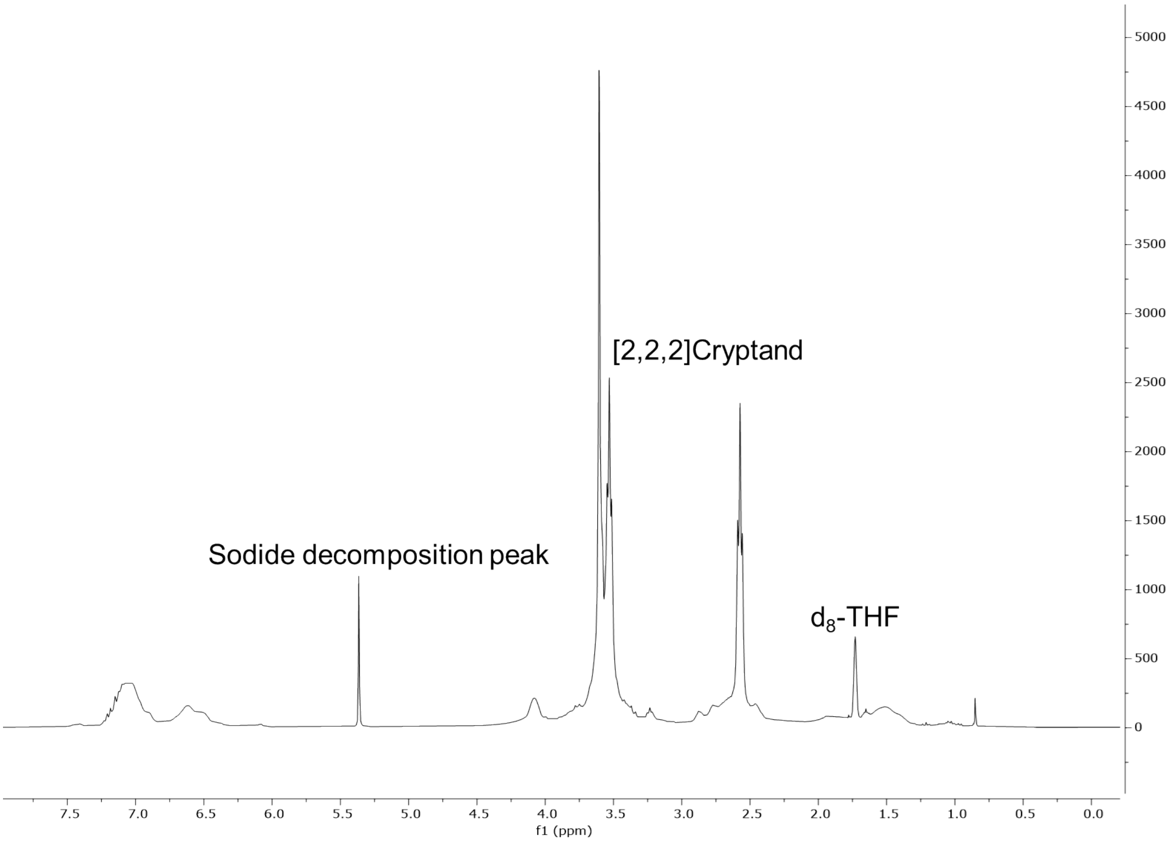

Figure S23. ^{1}H NMR (400 MHz, 25 °C) of an NMR scale reaction between **1** and styrene in d_8-THF.

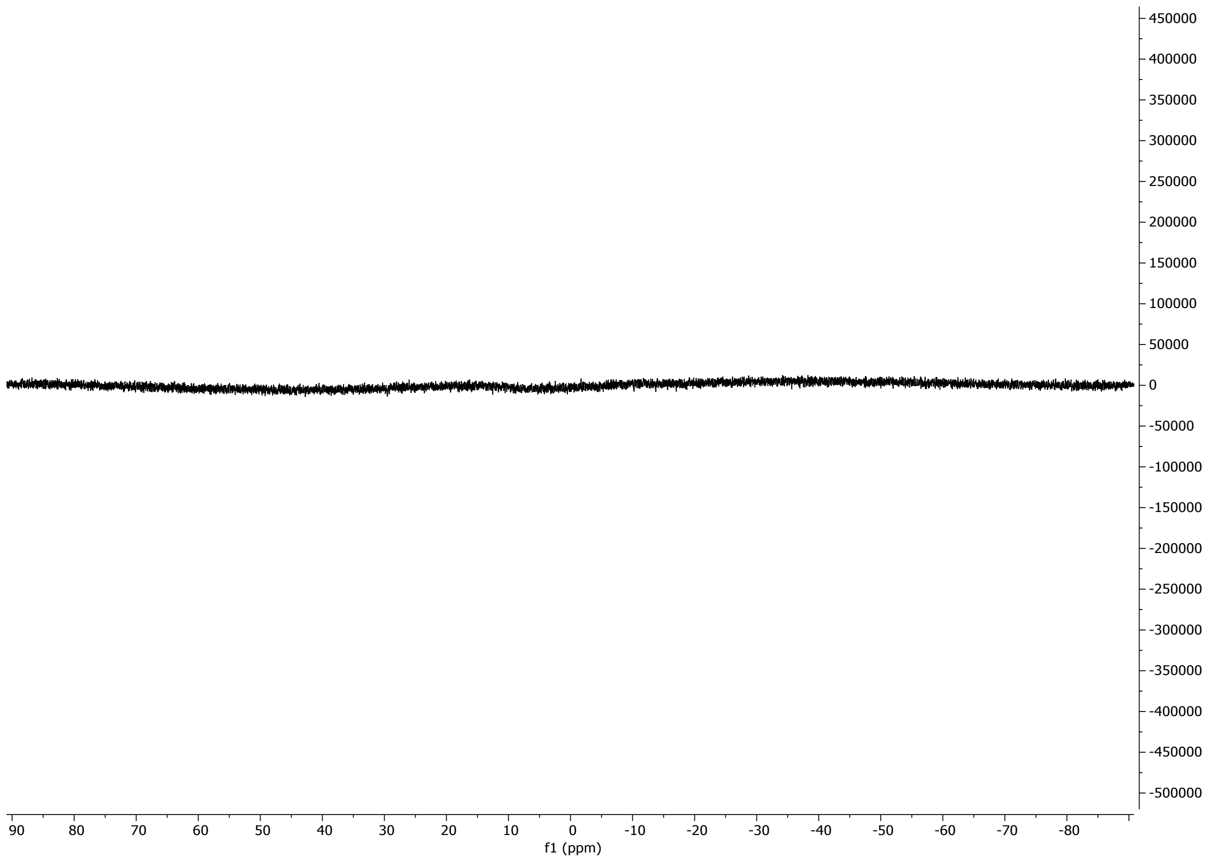

Figure S24. ^{23}Na NMR (106 MHz, 25 °C) of an NMR scale reaction between1 and styrene in d_8-THF.

1.2 Single Crystal X-Ray Diffraction

General Procedure:

Single crystal diffraction data for **2** were collected on a XtaLAB Synergy HyPix-Arc 100 diffractometer using copper radiation ($\lambda_{CuK\alpha}$ = 1.54184 Å). Data were collected at 150 K using an Oxford Cryosystems CryostreamPlus open-flow N_2 cooling device. Intensities were corrected for absorption using a multifaceted crystal model created by indexing the faces of the crystal for which data were collected[1]. Cell refinement, data collection and data reduction were undertaken via the software CrysAlisPro[2].

Single crystal diffraction data for **3**, **4**, **6** and **7** were measured on a Rigaku XtaLAB Synergy diffractometer using a HyPix detector while the dataset for **5** was measured on an Agilent SuperNova diffractometer using an Atlas detector. These data collections were driven and processed and absorption corrections were applied using CrysAlisPro.[3] The structures of **2** – **7** were solved using ShelXT[3] and refined by ShelXL[4] using the Olex2 interface[5]. All non-hydrogen atoms were refined with anisotropic displacement parameters. Unless otherwise stated, the hydrogen atoms were positioned with idealised geometry with the displacement parameters being constrained using a riding model with $U_{(H)}$ set to be an appropriate multiple of the U_{eq} value of the parent atom.

3: The structure contains two crystallographically-independent Na(Cryptand) and stilbene molecules per asymmetric unit. In one cryptand the group C(103)- C(104)/C(3B)- C(4B) is disordered over two positions at a refined percentage occupancy ratio of 69.3 (11) : 30.7 (11) and the atoms C(108), C(115)/C(8B), C(15B) are disordered over two positions at a refined percentage occupancy ratio of 83.0 (4) : 17.0 (4). The stilbene molecule C(201)-C(214) / C(1C)-C(14C) is disordered over two positions at a refined percentage occupancy ratio of 70.3 (6) : 29.7 (6). The stilbene molecule C(301)-C(314) / C(1D)-C(14D) is disordered over two positions at a refined percentage occupancy ratio of 73.9 (6) : 26.1 (6).

4: The cryptand group C(16)- C(18), O(4)/C(16A)- C(18A), O(4A) is disordered over two positions at a refined percentage occupancy ratio of 54.4 (10) : 45.6 (10) and the group C(7)- C(9), O(6)/C(7A)-C(9A), O(6A) is disordered over two positions at a refined percentage occupancy ratio of 59.3 (8) : 40.7 (8).

5: The structure contains two crystallographically-independent Na(Cryptand) units per asymmetric unit. In one cryptand the group C(8)-O(5)-C(15)/C(8A)-O(5A)-C(15A) is disordered over two positions at a refined percentage occupancy ratio of 62.4 (8) : 37.6 (8). In the second cryptand the group C(108)-O(105)-C(115)-C(116)/C(8B)-O(5B)-C(15B) -C(16B) is disordered over two positions, at a refined percentage occupancy ratio of 49.7 (6) : 50.3 (6), as are the oxygen atoms O(104), O(106) / O(4B), O(6B) at a refined percentage occupancy ratio of 58.8 (6) : 41.2 (6). The structure contains one molecule of tetrahydrofuran per asymmetric unit, disordered over two positions at a refined percentage occupancy ratio of 59.5 (12) : 40.5 (12).

6: The structure is composed of one positively charged Na(Cryptand) complex to half a doubly-negatively charged $[Na(C_8H_8)(THF)]_2$ complex with half a molecule of un-bound THF. The $[Na(C_8H_8)(THF)]_2$ complex is located on an inversion centre such that only half is crystallographically-unique. The THF ligand in this complex (O(7),C(27)-C(30) / O(7A),C(27A)-C(30A) / O(7B),C(27B)-C(30B)) is disordered over three positions at a percentage occupancy ratio of 0.5 : 0.25 : 0.25 respectively. The structure contains one molecule of un-bound THF per $[Na(C_8H_8)(THF)]_2$ complex, disordered over two positions across a 2-fold rotation axis, (at a 50 : 50 percentage occupancy ratio by symmetry).

The hydrogen atoms bound to the C_8H_8 unit were located in the electron density and their positions refined subject to appropriate geometric restraints. The remaining hydrogen atoms were fixed as riding models with the isotropic thermal parameters (U_{iso}) of all hydrogen atoms in the structure being based on the U_{eq} of the parent atom.

7: The hydrogen atoms bound to the C_8H_8 unit were located in the electron density and their positions and isotropic thermal parameters were refined subject to appropriate geometric restraints. The remaining hydrogen atoms were fixed as riding models with the isotropic thermal parameters (U_{iso}) being based on the U_{eq} of the parent atom.

SCXRD data tables

Table S1. Crystal Data and Refinement Details for **2-4**

	**2**	**3**	**4**
Empirical formula	$C_{64}H_{96}N_4Na_2O_{12}$	$C_{32}H_{48}N_2NaO_6$	$C_{38}H_{53}N_2NaO_6$
Formula weight	1159.42	579.71	656.81
Temperature/K	150.0(2)	100.00(10)	99.98(10)
Crystal system	monoclinic	monoclinic	monoclinic
Space group	$P2_1/n$	$P2_1/n$	$P2_1/n$
a/Å	15.4699(5)	12.46660(10)	14.6288(2)
b/Å	18.5991(7)	24.2183(2)	14.8933(2)
c/Å	21.3746(7)	21.04720(10)	16.0940(2)
α/°	90	90	90
β/°	95.097(3)	100.6350(10)	91.5480(10)
γ/°	90	90	90
Volume/Å^3	6125.7(4)	6245.41(8)	3505.14(8)
Z	4	8	4
ρ_{calc}g/cm^3	1.257	1.233	1.245
μ/mm^{-1}	0.812	0.797	0.772
F(000)	2504.0	2504.0	1416.0
Crystal size/mm^3	0.09 × 0.08 × 0.06	0.31 × 0.14 × 0.09	0.3 × 0.08 × 0.04
Radiation	CuKα (λ = 1.54184)	Cu Kα (λ = 1.54184)	Cu Kα (λ = 1.54184)
2Θ range for data collection/°	6.31 to 154.334	7.3 to 158.722	8.06 to 155.958
Index ranges	-18 ≤ h ≤ 19, -22 ≤ k ≤ 22, -23 ≤ l ≤ 26	-15 ≤ h ≤ 15, -30 ≤ k ≤ 29, -26 ≤ l ≤ 16	-18 ≤ h ≤ 16, -14 ≤ k ≤ 18, -20 ≤ l ≤ 19
Reflections collected	45050	60047	21331
Independent reflections	11919 [R_{int} = 0.0315, R_{sigma} = 0.0306]	12445 [R_{int} = 0.0361, R_{sigma} = 0.0277]	6756 [R_{int} = 0.0215, R_{sigma} = 0.0219]
Data/restraints/parameters	11919/0/739	12445/2578/1031	6756/296/507
Goodness-of-fit on F^2	1.031	1.027	1.083

Final R indexes [I>=2σ (I)]	R_1 = 0.0374, wR_2 = 0.0916	R_1 = 0.0530, wR_2 = 0.1399	R_1 = 0.0686, wR_2 = 0.1874
Final R indexes [all data]	R_1 = 0.0502, wR_2 = 0.0975	R_1 = 0.0616, wR_2 = 0.1465	R_1 = 0.0786, wR_2 = 0.1961
Largest diff. peak/hole / e $Å^{-3}$	0.25/-0.21	0.66/-0.41	0.80/-0.36

Table S2. Crystal Data and Refinement Details for **5-7**

	**5**	**6**	**7**
Empirical formula	$C_{66}H_{100}N_4Na_2O_{13}$	$C_{64}H_{110}N_4Na_4O_{15}$	$C_{30}H_{52}N_2Na_2O_7$
Formula weight	1203.47	1267.51	598.71
Temperature/K	125.00(10)	100.00(10)	100.00(10)
Crystal system	monoclinic	monoclinic	monoclinic
Space group	$P2_1/c$	C2/c	$P2_1/n$
a/Å	11.9585(4)	29.7845(4)	9.27640(10)
b/Å	36.1695(11)	11.9146(2)	18.5126(2)
c/Å	15.7212(5)	20.8353(2)	18.7295(2)
α/°	90	90	90
β/°	111.566(4)	91.7980(10)	103.8280(10)
γ/°	90	90	90
Volume/$Å^3$	6323.9(4)	7390.19(17)	3123.20(6)
Z	4	4	4
$\rho_{calc}g/cm^3$	1.264	1.139	1.273
μ/mm^{-1}	0.817	0.846	0.959
F(000)	2600.0	2744.0	1296.0
Crystal size/mm^3	0.35 × 0.08 × 0.07	0.18 × 0.12 × 0.06	0.128 × 0.123 × 0.076
Radiation	Cu Kα (λ = 1.54184)	Cu Kα (λ = 1.54184)	Cu Kα (λ = 1.54184)
2Θ range for data collection/°	7.776 to 156.426	8.492 to 155.656	6.814 to 158.758
Index ranges	$-15 \leq h \leq 14$, $-45 \leq k \leq 45$, $-19 \leq l \leq 15$	$-36 \leq h \leq 37$, $-14 \leq k \leq 13$, $-20 \leq l \leq 26$	$-11 \leq h \leq 9$, $-22 \leq k \leq 22$, $-22 \leq l \leq 23$

Reflections collected	45653	32105	24798
Independent reflections	13196 [R_{int} = 0.0453, R_{sigma} = 0.0484]	7227 [R_{int} = 0.0328, R_{sigma} = 0.0261]	6232 [R_{int} = 0.0315, R_{sigma} = 0.0255]
Data/restraints/parameters	13196/268/896	7227/942/529	6232/0/402
Goodness-of-fit on F^2	1.011	1.041	1.060
Final R indexes [I>=2σ (I)]	R_1 = 0.0534, wR_2 = 0.1335	R_1 = 0.0913, wR_2 = 0.2817	R_1 = 0.0344, wR_2 = 0.0883
Final R indexes [all data]	R_1 = 0.0802, wR_2 = 0.1539	R_1 = 0.1035, wR_2 = 0.2985	R_1 = 0.0391, wR_2 = 0.0909
Largest diff. peak/hole / e $Å^{-3}$	0.38/-0.28	0.86/-0.77	0.30/-0.30

Section 2. Computational details and data

2.1 General Procedure:

Geometry optimisations and vibrational frequency calculations were performed for each structure considered in this work, using experimental SCXRD geometries as starting points where available. Initial optimisations and frequency calculations were carried out at the B3LYP/6-31G(d,p) level of theory[7,8]. Solvent effects were included via the integral equation formalism of the polarizable continuum model (IEFPCM), using tetrahydrofuran (THF) as the solvent. Grimme's D3 dispersion correction with Becke–Johnson damping (GD3BJ) was also applied[9]. Further single point energy calculations, were performed at the B3LYP/6-311++G(d,p) level to obtain more accurate natural population analysis (NPA) charges and molecular orbital (MO) diagrams[10]. Both the solvent model and dispersion correction were used consistently in the geometry optimisations and the subsequent single point energy (SPE) calculations, as implemented in Gaussian 16[11]. All calculations, including geometry optimisations, frequency analysis, and natural bond orbital (NBO) charge analysis, were performed using the Gaussian 16 C.02-AVX2 package[10,11]. Molecular orbital diagrams and spin density plots were generated using ChemCraft 1.8, based on the optimised geometry but using the wavefunction from the higher-level SPE calculation[12].

2.2 Molecular orbital diagrams of **2** to **7**

The following molecular orbital diagrams are based on the optimised minimum structures of **2-7** are show in Figures S26-29 respectively.

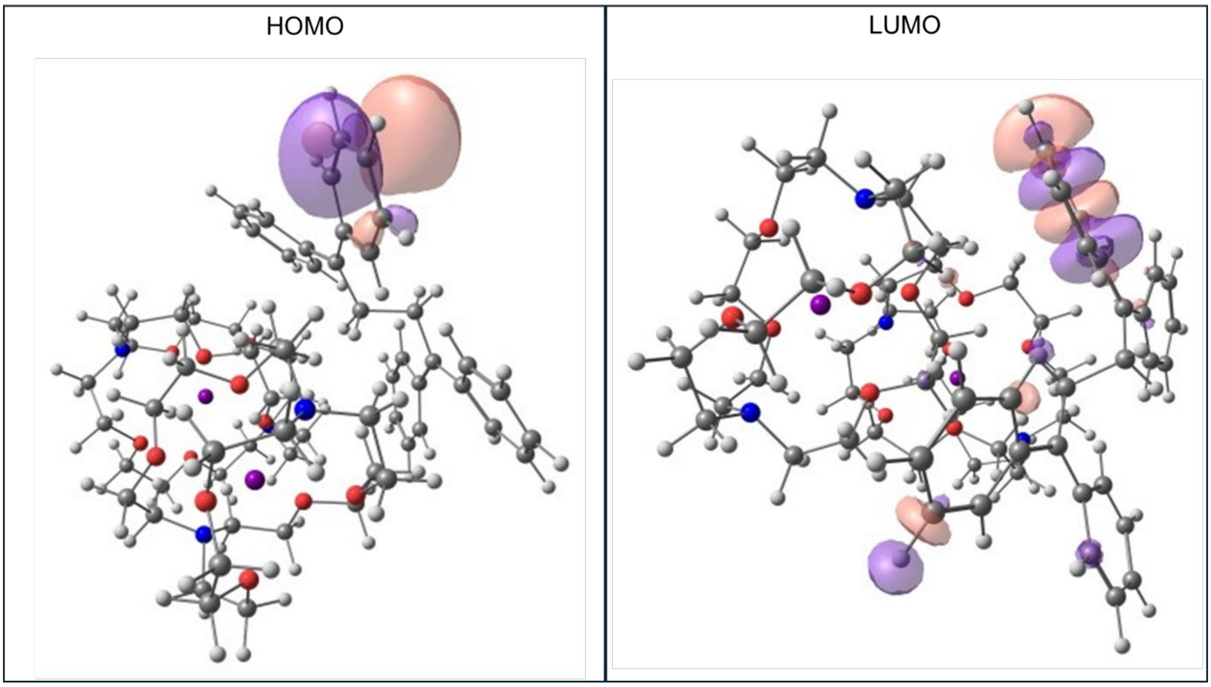

Figure S25. Diagrams of the HOMO (left) and LUMO (right) of the optimised structure of **2** generated using Chemcraft 1.8 with a contour value of 0.02**.**

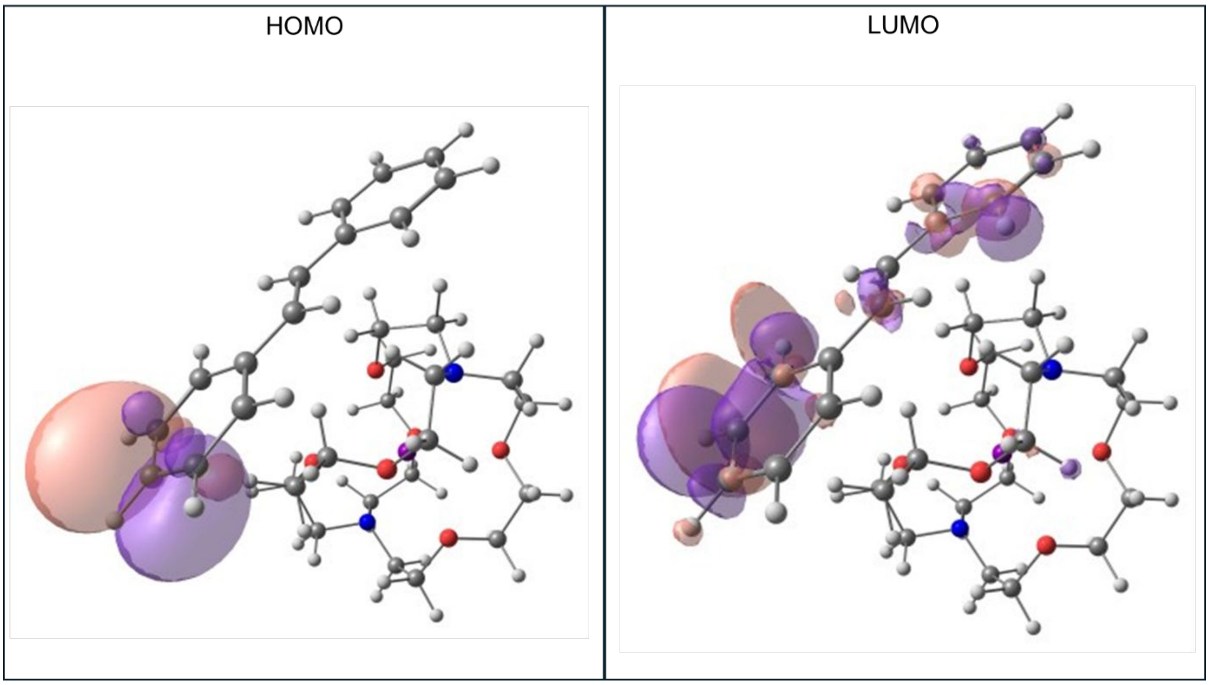

Figure S26. Diagrams of the HOMO (left) and LUMO (right) of the optimised structure of **3** generated using Chemcraft 1.8 with a contour value of 0.02**.**

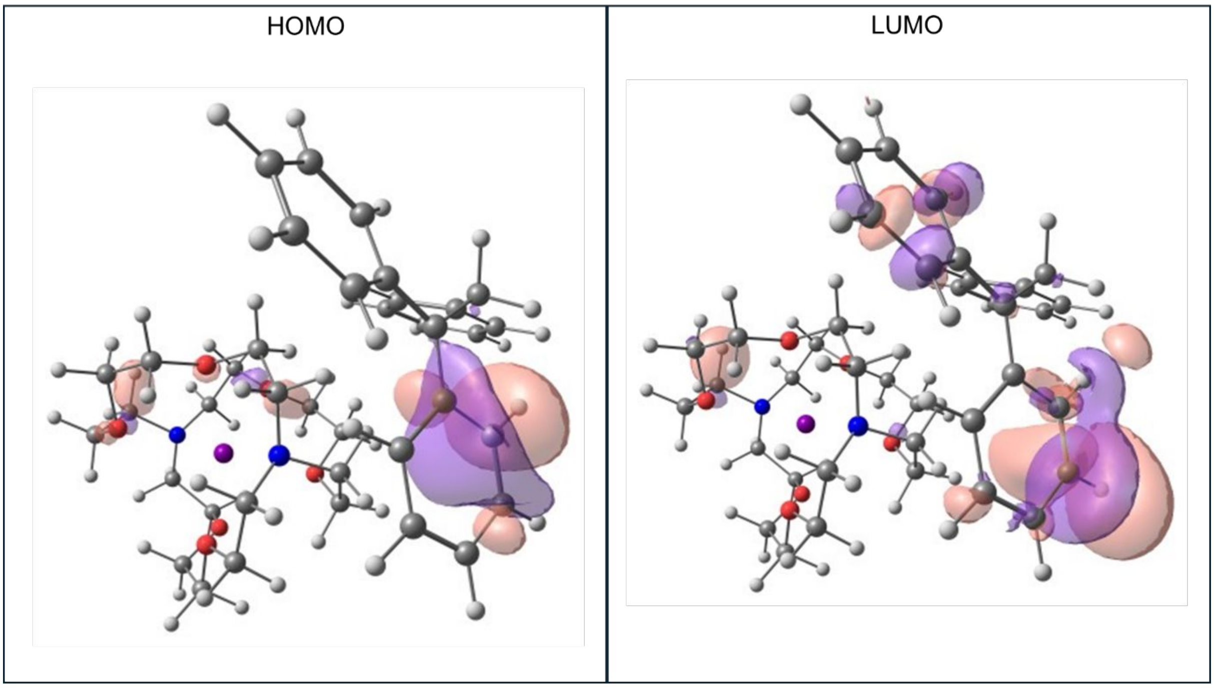

Figure S27. Diagrams of the HOMO (left) and LUMO (right) of the optimised structure of **4** generated using Chemcraft 1.8 with a contour value of 0.02**.**

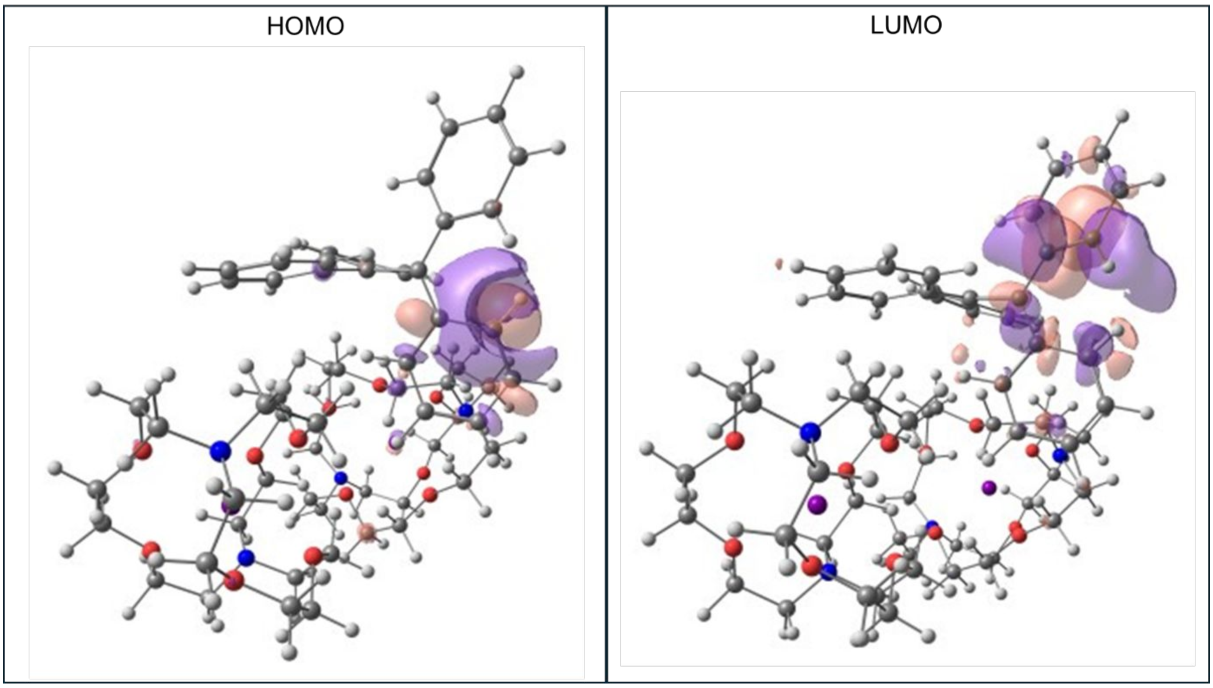

Figure S28. Diagrams of the HOMO (left) and LUMO (right) of the optimised structure of **5** generated using Chemcraft 1.8 with a contour value of 0.02**.**

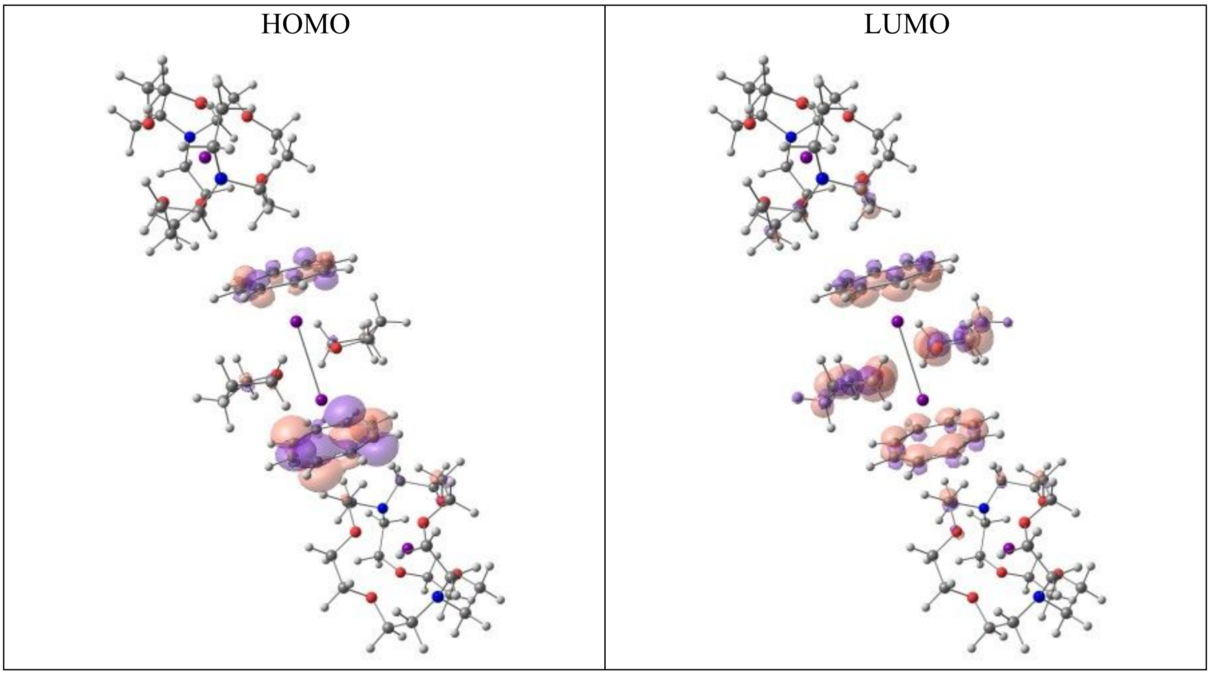

Figure S29. Diagrams of the HOMO (left) and LUMO (right) of the optimised structure of **6** generated using Chemcraft 1.8 with a contour value of 0.02**.**

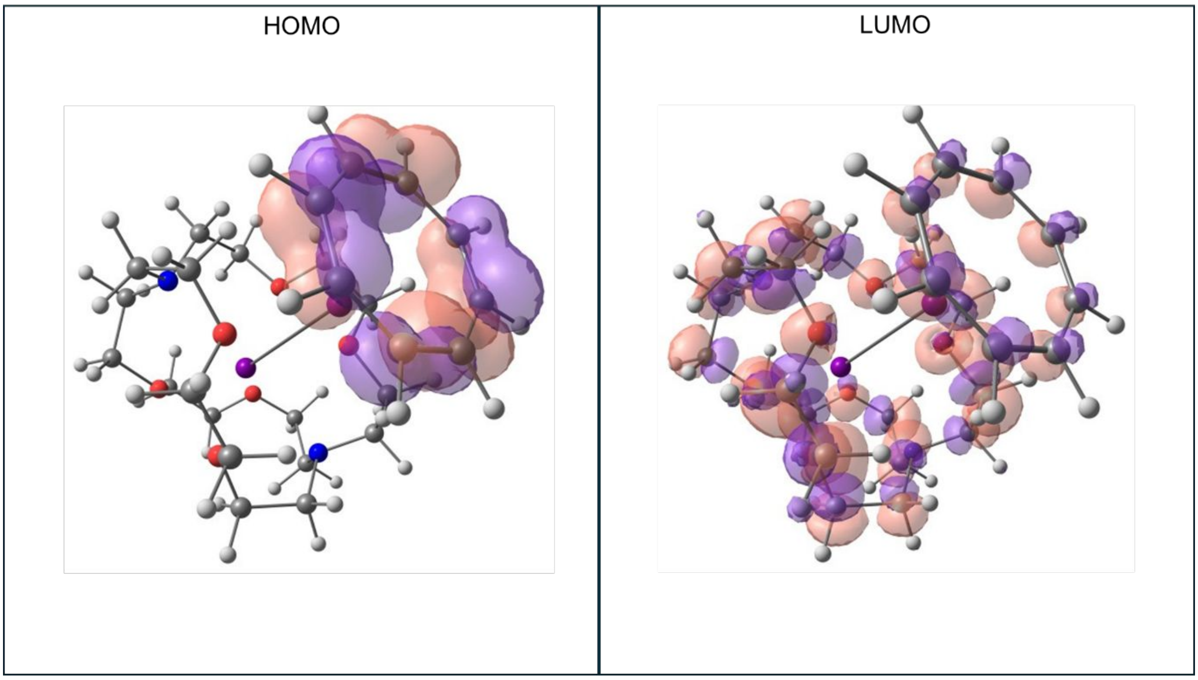

Figure S30. Diagrams of the HOMO (left) and LUMO (right) of the optimised structure of **7** generated using Chemcraft 1.8 with a contour value of 0.02**.**

The frontier molecular orbitals of each species considered in this work exhibit anion localised HOMO orbitals, suggesting that highest energy pair of electrons is the source of the negative charge. Interestingly, for products (2, 3, 4 and 5) the LUMO is also based on the anionic species, signifying a high degree of charge separation between the two components of the SIP. Whereas products 6 and 7 still exhibit anion based HOMO orbitals, their respective LUMOs are now spread slightly among the coordinating THF molecules (6) and the 2,2,2-crpytand (7) suggesting a larger degree of interaction between the charged species in these two structures.

2.3 NPA charges of **2, 3, 4, 5, 6 and 7**

The NPA charges of the anionic components of the products are detailed below highlighting their overall charge. The [Na^+(2,2,2-cryptand)] counter ions are not shown as they are consistent across all the structures to within a small degree of variance to those shown in Figure 6 in the main text.

(a)

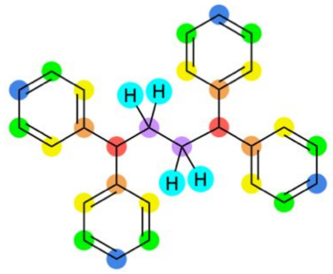

Atom	NPA charges / e	Average NPA charge / e
C	-0.400, -0.381	-0.391
C	-0.227, -0.241	-0.234
C_{ipso}	-0.077, -0.045, -0.048, -0.034	-0.051
C_{ortho}	-0.172, -0.273, -0.291, -0.277, -0.285, -0.260, -0.277, -0.272	-0.263
C_{meta}	-0.259, -0.219, -0.160, -0.218, -0.216, -0.221, -0.171, -0.206	-0.209
C_{para}	-0.334, -0.328, -0.350, -0.330	-0.336
H	0.194, 0.173, 0.185, 0.195	0.187
H_{Ph}	0.186, 0.193, 0.197, 0.193, 0.198, 0.201, 0.190, 0.197, 0.194, 0.198, 0.190, 0.193, 0.195, 0.195, 0.201, 0.198, 0.186, 0.192, 0.190, 0.199	0.194
Total	-1.939	

(b)

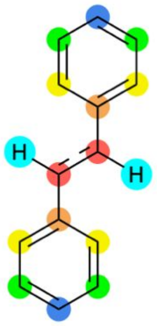

Atom	NPA charges / e	Average NPA charge / e
C	-0.351, -0.298	-0.325
C_{ipso}	-0.102, 0.025	-0.039
C_{ortho}	-0.296, -0.271, -0.233, -0.269	-0.267
C_{meta}	-0.228, -0.200, -0.228, -0.215	-0.218
C_{para}	-0.315, -0.307	-0.311
H	0.181, 0.182	0.182
H_{Ph}	0.193, 0.198, 0.198, 0.197, 0.196, 0.194, 0.198, 0.199, 0.197, 0.195	0.197
Total	-0.959	

(c)

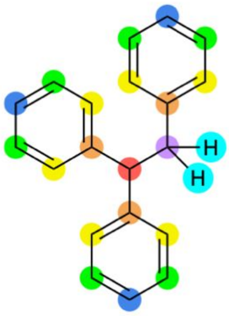

Atom	NPA charges / e	Average NPA charge / e
C	-0.408	-0.408
C	-0.242	-0.242
C_{ipso}	-0.044, -0.050, -0.006	-0.033
C_{ortho}	-0.224, -0.218, -0.268, -0.275, -0.290, -0.265	-0.257
C_{meta}	-0.222, -0.208, -0.212, -0.220, -0.137, -0.214	-0.202
C_{para}	-0.231, -0.325, -0.336	-0.307
H	0.198, 0.218	0.208
H_{Ph}	0.214, 0.207, 0.206, 0.209, 0.205, 0.196, 0.196, 0.198, 0.195, 0.202, 0.201, 0.191, 0.197, 0.196, 0.195	0.201
Total	-0.973	

(d)

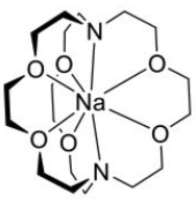

[Na^+(2,2,2-cryptand)] #1

Atom	NPA charges / e	Average NPA charge / e
Na	0.831	0.831
O	-0.636, -0.633, -0.633, -0.637, -0.627, -0.653	-0.637
C	-0.036, -0.041, -0.032, -0.187, -0.189, -0.023, -0.041, -0.038, -0.035, -0.186, -0.172, -0.037, -0.221, -0.051, -0.030, -0.053, -0.021, -0.179	-0.084
N	-0.600, -0.598	-0.599
H	0.175, 0.213, 0.186, 0.164, 0.173, 0.213, 0.198, 0.205, 0.172, 0.195, 0.159, 0.167, 0.209, 0.213, 0.226, 0.185, 0.173, 0.187, 0.167, 0.190, 0.178, 0.199, 0.178, 0.178, 0.177, 0.202, 0.187, 0.184, 0.173, 0.196, 0.198, 0.181, 0.179, 0.182, 0.185, 0.175	0.187
Total	0.964	

[Na^+(2,2,2-cryptand)] #2

Atom	NPA charges / e	Average NPA charge / e
Na	0.833	0.833
O	-0.627, -0.632, -0.630, -0.624, -0.628, -0.644	-0.631
C	-0.208, -0.032, -0.086, -0.043, -0.036, -0.181, -0.200, -0.177, -0.024, -0.033, -0.041, -0.038, -0.035, -0.025, -0.193, -0.192, -0.033, -0.033	-0.089
N	-0.604, -0.599	-0.602
H	0.190, 0.197, 0.176, 0.184, 0.187, 0.177, 0.185, 0.175, 0.181, 0.197, 0.181, 0.187, 0.203, 0.182, 0.218, 0.180, 0.179, 0.172, 0.203, 0.172, 0.180, 0.183, 0.183, 0.183, 0.206, 0.202, 0.193, 0.223, 0.172, 0.193, 0.192, 0.179, 0.177, 0.185, 0.171, 0.178	0.187
Total	0.961	

(e)

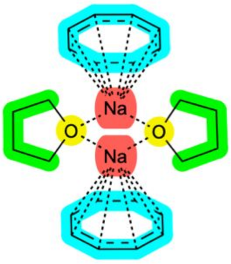

Atom	NPA charges / e	Average NPA charge / e
Na	0.906, 0.909	0.908
C_{COT}	-0.410, -0.417, -0.413, -0.410, -0.408, -0.419, -0.429, -0.412, -0.410, -0.424, -0.425, -0.406, -0.410, -0.418, -0.412, -0.411	-0.415
O	-0.684, -0.685	-0.685
C_{THF}	-0.411, -0.410, -0.410, -0.410, -0.029, -0.031, -0.028, -0.032	-0.220
H_{COT}	0.176, 0.178, 0.178, 0.178, 0.178, 0.178, 0.178, 0.178, 0.178, 0.178, 0.178, 0.177, 0.177, 0,177, 0.177, 0.180	0.178
H_{THF}	0.209, 0.210, 0.210, 0.206, 0.199, 0.178, 0.181, 0.200, 0.181, 0.200, 0.197, 0.181, 0.209, 0.208, 0.208, 0.209	0.199
Total	-1.919	

(f)

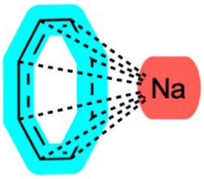

Atom	NPA charges / e	Average NPA charge / e
Na	0.878	0.878
C	-0.415, -0.411, -0.413, -0.419, -0.421, -0.409, -0.412, -0.418	-0.415
H	0.177, 0.177, 0.179, 0.179, 0.177, 0.177, 0.179, 0.178	0.178
Total	-1.017	

Figure S31. NPA charge breakdown for the anionic components of structures **2, 3, 4, 5** (the two counter cations ([Na^+(2,2,2-cryptand)]), **6 and 7** (a, b, c, d, e and f respectively), with separate and total charges shown in the table alongside.

2.4 Free energy of formation calculations of **6** and **7**

The free energy of formation ($\Delta G_{formation}$) of both products **6** and **7** were calculated using equation 1, with the energies used in the calculations (with BS-A and BS-B referring to the optimisation and SPE basis sets respectively) outlined in Table S3 (all energies provided in $kcalmol^{-1}$).

$$\Delta G_{formation} = \sum G_{products} - \sum G_{reactants} \qquad \text{(Equation 1)}$$

	EE (BS-1)	G_{corr} (BS-1)	EE (BS-2)	G = EE (BS-2) + Gcorr (BS-1)	$\Delta G_{formation}$
**1**	-999727	313.9208	-999924	-999610	-
COT	-194294	64.56754	-194340	-194276	-
THF	-145882	55.98196	-145920	-145864	-
**6**	-2679991	936.1518	-2680551	-2679615	-117.176
**7**	-1194094	394.0091	-1194344	-1193950	-64.3046

It is clear that both products are thermodynamically favourable to form, signified by the negative value of formation energy. As there are two equivalents of COT and [Na^+(2,2,2-cryptand)Na^-] in the formation of product **6**, to directly compare the two the $\Delta G_{formation}$ of product **7** was doubled, yielding -128.609 $kcalmol^{-1}$. This suggests that it is more thermodynamically favourable to form two instances product **7** rather than **6**.

2.5 Atomic coordinates of optimized structures

1

Na	-0.23705600	0.11763800	-0.00698500
O	-0.68492300	2.58600600	-0.13991000
O	1.15729600	-0.49197900	-1.97610000
O	1.04182400	-0.25479500	2.07222600
N	2.08245300	1.54240700	-0.02338700
C	1.74017000	2.94028500	-0.30874400
H	1.66060300	3.06442500	-1.39146200
H	2.53260500	3.63428800	0.02151500
C	0.41925600	3.35606000	0.33174400
H	0.44354100	3.19645400	1.41271300
H	0.24014600	4.42570800	0.15454200
C	2.92572600	0.96143700	-1.07724300
H	3.46653100	0.09854300	-0.66880600
H	3.70588500	1.66849300	-1.41009900
C	2.11715600	0.51480000	-2.29011000
H	1.54133900	1.34506400	-2.70894400
H	2.80314900	0.15459700	-3.06996500
C	2.69637600	1.38990600	1.30401900
H	2.28860600	2.15725600	1.96716300
H	3.78697400	1.55587400	1.26923400
C	2.43496900	0.01466600	1.90646000
H	2.82579600	-0.77925700	1.26015900
H	2.95636300	-0.07314500	2.87005700
C	-1.17554400	2.97825600	-1.41552700
H	-0.38878200	2.92336200	-2.17928800
H	-1.55300000	4.01010400	-1.38214600
C	1.71828400	-1.79556900	-1.83829100
H	2.52122600	-1.81862600	-1.08504900
H	2.15465900	-2.12715700	-2.79220700
C	0.45440300	0.35269600	3.21357700
H	0.55172100	1.44645600	3.17862300
H	0.93790500	-0.00589600	4.13320400
O	-1.60833800	0.54353900	2.06715700
O	0.12435600	-2.30562100	-0.16298200
O	-1.73747000	0.73332400	-1.90481400
N	-2.64534400	-1.31918900	0.01630900
C	-3.23450900	-1.15065900	1.34879600
H	-2.78545900	-1.88507400	2.02171300
H	-4.31915000	-1.35603600	1.34912200
C	-2.99278100	0.24633500	1.91348800
H	-3.38237700	1.01160400	1.23722700
H	-3.51533800	0.35232500	2.87436300
C	-2.28780900	-2.71676400	-0.25536200
H	-2.23163000	-2.85694500	-1.33765100
H	-3.06088900	-3.41655000	0.10609500
C	-0.94164900	-3.09179200	0.35787100
H	-0.94084100	-2.91241300	1.43628600
H	-0.74372500	-4.16032500	0.19725500
C	-3.49108900	-0.75099100	-1.03882000
H	-4.03270800	0.10500800	-0.62899500
H	-4.26001000	-1.46535600	-1.38044700
C	-2.67321200	-0.28738500	-2.24097500
H	-2.07464800	-1.10833300	-2.64368200
H	-3.34662300	0.05966000	-3.03685900
C	-1.00906100	-0.02045600	3.22816900
H	-1.10583900	-1.11413100	3.23411400
H	-1.48699500	0.37380000	4.13615400
C	0.61491100	-2.73813500	-1.42805400
H	-0.18391900	-2.73570400	-2.18226700
H	1.01888400	-3.75779400	-1.35173300
C	-2.29630300	2.03485900	-1.78368800
H	-3.07066200	2.06578100	-1.00569800
H	-2.74973700	2.34883600	-2.73452000
Na	5.44423000	-2.47186800	0.09529000

2

Na	2.5967	2.53925	-0.20601
O	3.50131	4.46779	0.89321
O	1.05249	3.64003	-1.90762
O	1.50372	1.76457	2.08379
O	1.61344	0.94514	-1.7758
O	4.10775	1.2926	1.33867
O	4.11807	3.92758	-1.72837
N	0.52111	4.17955	0.94294
N	4.53124	1.00087	-1.56423
C	1.26973	5.29018	1.5473
H	0.73961	5.69669	2.42639
H	1.3206	6.10649	0.82155
C	2.6998	4.94483	1.96901
H	3.16217	5.8344	2.41924
H	2.71825	4.14747	2.70985
C	-0.34819	4.63651	-0.14703
H	-0.86537	5.5815	0.09727
H	-1.13427	3.89291	-0.28837
C	0.4169	4.82867	-1.45269
H	-0.26635	5.21422	-2.22389
H	1.22207	5.55725	-1.3286
C	-0.23664	3.38552	1.91489
H	-0.86696	2.68266	1.36536
H	-0.90413	4.01808	2.5309
C	0.6315	2.57201	2.85681
H	1.20805	3.20808	3.54232
H	-0.0225	1.95034	3.483
C	4.07264	5.47008	0.06307
H	4.67563	6.16644	0.66209
H	3.29621	6.04511	-0.45923
C	4.95334	4.76814	-0.94512
H	5.45894	5.51082	-1.57884
H	5.71968	4.18652	-0.41517
C	0.1666	2.67758	-2.46421
H	-0.37432	3.10657	-3.32119
H	-0.56312	2.32359	-1.72855
C	0.9899	1.50109	-2.92359
H	0.33691	0.75699	-3.39994
H	1.73509	1.82921	-3.66009
C	2.34462	0.92809	2.87417
H	1.75083	0.1661	3.39608

H	2.87385	1.53506	3.62338
C	3.34068	0.25814	1.96113
H	3.98257	-0.4124	2.54153
H	2.81528	-0.34881	1.21351
C	2.43174	-0.19516	-2.04983
H	1.93061	-0.87018	-2.75205
H	2.51294	-0.72628	-1.10145
C	3.80623	0.20481	-2.56344
H	4.35698	-0.70335	-2.85184
H	3.69015	0.79904	-3.47318
C	5.34503	0.85789	0.77833
H	5.85972	0.18823	1.47841
H	5.94533	1.76514	0.66174
C	5.16532	0.14456	-0.5551
H	6.14387	-0.2376	-0.89581
H	4.54018	-0.73514	-0.38978
C	4.80014	3.17787	-2.73068
H	5.54176	3.80957	-3.23931
H	4.03446	2.9095	-3.46284
C	5.4827	1.92999	-2.1771
H	6.06337	1.45414	-2.98752
H	6.20921	2.23333	-1.41905
Na	-3.77782	0.59115	0.19057
O	-3.62364	3.15133	-0.46311
O	-1.99647	0.20513	-1.52531
O	-6.07003	-0.13173	-0.8282
O	-4.23481	-1.86069	0.3232
O	-4.91789	2.1604	1.81922
O	-1.58336	0.25002	1.24642
N	-4.37011	1.38056	-2.72145
N	-3.9074	-0.34553	2.76518
C	-4.46074	2.83721	-2.76226
H	-5.4688	3.13325	-2.46067
H	-4.31537	3.23896	-3.78174
C	-3.44525	3.49408	-1.83628
H	-2.4405	3.16171	-2.08904
H	-3.48191	4.58609	-1.95684
C	-3.22139	0.85102	-3.46567
H	-2.48267	1.64592	-3.59598
H	-3.50837	0.52477	-4.47792
C	-2.54566	-0.29794	-2.74125
H	-3.24543	-1.11666	-2.52871
H	-1.74629	-0.7166	-3.36738
C	-5.62954	0.73258	-3.08319
H	-5.41034	-0.27985	-3.43052
H	-6.13567	1.2422	-3.92207
C	-6.58672	0.65491	-1.89846
H	-6.76325	1.64756	-1.47577
H	-7.55529	0.25229	-2.22821
C	-4.69411	3.85336	0.15627
H	-5.66049	3.55474	-0.27105
H	-4.57454	4.9365	0.01023
C	-4.68114	3.54877	1.63183
H	-3.70913	3.82483	2.06666
H	-5.46234	4.14466	2.12541
C	-1.11392	-0.73512	-0.90131
H	-0.30913	-1.01883	-1.58863
H	-1.66321	-1.64839	-0.65034
C	-0.53332	-0.0859	0.3293
H	0.17789	-0.77285	0.79483
H	0.00373	0.82475	0.06588
C	-6.09686	-1.53253	-1.09368
H	-5.45858	-1.79009	-1.94736
H	-7.12364	-1.85717	-1.31445
C	-5.58501	-2.24788	0.12779
H	-6.20329	-1.9846	0.99641
H	-5.65138	-3.33364	-0.02895

C	-3.62953	-2.46514	1.46258
H	-2.55719	-2.38337	1.30731
H	-3.86962	-3.53627	1.49504
C	-4.07683	-1.80815	2.76724
H	-5.13525	-2.03143	2.92735
H	-3.52922	-2.27487	3.5964
C	-5.05229	1.83083	3.19803
H	-5.99273	2.24022	3.594
H	-4.23499	2.29294	3.7674
C	-5.06342	0.32273	3.36689
H	-5.1642	0.11258	4.44833
H	-5.96011	-0.06877	2.87694
C	-1.43427	-0.33565	2.53875
H	-0.52477	0.03335	3.02226
H	-1.32381	-1.42101	2.47062
C	-2.6362	0.06673	3.36914
H	-2.50941	-0.34483	4.38456
H	-2.62588	1.15635	3.44652
C	2.09822	-3.6502	2.078
C	0.99484	-3.45315	2.97321
C	-0.26141	-4.12873	2.78734
H	-0.37619	-4.81133	1.9544
C	-1.32931	-3.99319	3.66393
H	-2.23266	-4.57087	3.47561
C	-1.26872	-3.14035	4.77466
H	-2.10026	-3.0505	5.46642
C	-0.08997	-2.40102	4.94629
H	-0.01316	-1.68954	5.76718
C	0.98985	-2.54226	4.08688
H	1.85553	-1.91711	4.2579
C	3.44205	-3.21913	2.34441
C	3.96625	-2.94606	3.65435
H	3.34391	-3.1353	4.51938
C	5.26673	-2.51193	3.87336
H	5.59035	-2.33145	4.89723
C	6.17037	-2.33003	2.81653
H	7.18506	-1.98604	2.99104
C	5.71996	-2.65809	1.53105
H	6.39593	-2.56928	0.68234
C	4.42038	-3.08925	1.30126
H	4.14494	-3.33119	0.28452
C	1.79424	-4.26626	0.71829
H	1.15047	-5.15149	0.81066
H	2.70751	-4.64596	0.2515
C	1.10502	-3.26956	-0.25044
H	0.18213	-2.94856	0.23948
H	1.73947	-2.3697	-0.25293
C	0.79406	-3.70243	-1.67182
C	-0.58696	-3.90579	-2.02428
C	-1.11722	-3.68785	-3.33743
H	-0.44606	-3.3578	-4.12224
C	-2.46734	-3.81432	-3.63328
H	-2.80284	-3.61157	-4.64929
C	-3.40241	-4.17358	-2.65099
H	-4.45482	-4.29465	-2.88974
C	-2.92067	-4.39357	-1.35466
H	-3.61082	-4.68892	-0.56751
C	-1.57271	-4.25969	-1.04642
H	-1.25426	-4.45323	-0.02787
C	1.87894	-3.84939	-2.59526
C	3.20035	-3.37721	-2.29454
H	3.3727	-2.87294	-1.35449
C	4.27542	-3.52526	-3.16241
H	5.24594	-3.13368	-2.86059
C	4.13527	-4.16717	-4.39819
H	4.97612	-4.28438	-5.07479
C	2.86641	-4.67881	-4.71286

H	2.72127	-5.22018	-5.64667
C	1.78678	-4.53601	-3.85411
H	0.84598	-4.99685	-4.13102

3

C	-4.55187	-0.93995	-1.52723
H	-5.01722	-1.07977	-0.54816
H	-5.14764	-1.49661	-2.26404
C	-4.51686	0.53808	-1.90552
H	-3.99755	0.64459	-2.86077
H	-5.54907	0.89055	-2.0756
C	-2.63841	-1.83675	-2.65465
H	-2.53937	-0.95111	-3.29609
H	-3.23815	-2.58613	-3.19045
C	-1.26753	-2.39396	-2.35292
H	-1.36658	-3.26217	-1.68726
H	-0.79001	-2.7299	-3.28468
C	0.66308	0.38418	2.27042
H	-0.02471	0.09358	3.07005
H	1.45271	1.0064	2.71433
C	1.29672	-0.8372	1.61376
H	1.9507	-0.50648	0.80591
H	1.95478	-1.33642	2.34439
C	0.66952	1.97923	0.4951
H	1.37755	1.37698	-0.08095
H	1.25097	2.7063	1.0746
C	-0.26608	2.7003	-0.43903
H	-1.00004	3.27879	0.13864
H	0.31286	3.39225	-1.06406
C	0.81456	-1.75789	-1.35757
H	1.3887	-0.83472	-1.25623
H	1.28236	-2.35156	-2.15172
C	0.82836	-2.54672	-0.05478
H	0.21012	-3.44054	-0.17509
H	1.85314	-2.89256	0.13969
C	-3.87968	1.86743	1.52467
H	-3.11638	2.63877	1.39543
H	-4.54932	2.18896	2.33442
C	-4.67984	1.67458	0.23978
H	-5.38949	0.85656	0.38595
H	-5.28659	2.57769	0.05393
C	-3.96327	-0.29567	2.55094
H	-4.78334	-0.63352	1.90335
H	-4.39819	0.10421	3.47787
C	-3.06148	-1.46423	2.87088
H	-2.22367	-1.11682	3.48936
H	-3.61964	-2.22154	3.43979
C	-1.87035	2.24362	-2.15929
H	-1.98755	1.48257	-2.93563
H	-1.4842	3.15314	-2.64016
C	-3.20779	2.54738	-1.48933
H	-3.04263	3.27703	-0.69309
H	-3.8771	3.03431	-2.21973
C	-1.68777	-3.09004	1.77382
H	-1.70985	-3.60887	0.81214
H	-2.04241	-3.79176	2.54171
C	-0.27319	-2.62656	2.10934
H	-0.29844	-2.07026	3.04972
H	0.35862	-3.51292	2.29494
N	-3.81891	1.34905	-0.90247
N	0.29046	-1.76094	1.06926
O	-3.25058	-1.5073	-1.41308
O	-0.11698	1.15971	1.36193
O	-0.50583	-1.36212	-1.74549
O	-3.17005	0.69283	1.90721
O	-0.91287	1.71911	-1.24783
O	-2.60388	-2.00444	1.63826
Na	-1.70886	-0.21912	0.08847
C	4.04875	0.02292	-0.72435
H	3.81404	0.35894	-1.73348
C	4.15197	0.98623	0.28426
H	4.42971	0.65355	1.28356
C	4.21454	-1.38633	-0.583
C	4.08662	-2.24757	-1.7163
H	3.89697	-1.79473	-2.68751
C	4.17814	-3.62604	-1.61192
H	4.0645	-4.23571	-2.50594
C	4.41665	-4.24378	-0.37082
H	4.48828	-5.32367	-0.28859
C	4.57294	-3.4215	0.75576
H	4.76756	-3.87279	1.72685
C	4.48073	-2.0399	0.66294
H	4.5959	-1.44286	1.562
C	3.8916	2.38121	0.16497
C	3.99272	3.23644	1.30526
H	4.31888	2.80057	2.24795
C	3.67371	4.58372	1.24982
H	3.76201	5.18896	2.14969
C	3.23805	5.17581	0.05005
H	2.98762	6.23099	0.00614
C	3.14426	4.36299	-1.09015
H	2.81641	4.79688	-2.03312
C	3.4613	3.01224	-1.04637
H	3.36229	2.41856	-1.94992

4

Na	2.35992	0.21307	-0.15152
O	3.40974	2.33397	1.05875
O	1.02706	1.11209	1.68404
O	2.03473	-1.66441	1.41888
O	4.18882	-0.4755	-1.7288
O	2.37125	1.48473	-2.29643
N	4.70979	-0.34417	1.16215
N	-0.09761	0.79917	-1.00146
C	1.87816	-2.92631	0.78021
H	2.83128	-3.28221	0.36746
C	0.87602	-2.73883	-0.33501
H	0.70276	-3.69656	-0.84739
H	-0.0767	-2.40333	0.08235
C	5.0334	0.78402	2.04432
H	6.0807	0.74141	2.39192
H	4.40944	0.70962	2.93831
C	4.78265	2.13826	1.38648
H	5.13786	2.93861	2.04949
H	5.3327	2.22758	0.44624
C	2.62069	2.77507	2.16245
H	2.78543	3.84615	2.3442
H	2.88634	2.22566	3.0741
C	1.1703	2.51523	1.83538
H	0.53116	2.89078	2.64729
H	0.89918	3.04577	0.91371
C	-0.31021	0.65298	1.47578
H	-0.94981	0.95887	2.31739
H	-0.22214	-0.43308	1.48141
C	-0.93276	1.13686	0.17296
H	-1.93865	0.70997	0.09823
H	-1.07552	2.21953	0.21603
C	0.06095	1.96682	-1.87881
H	-0.87831	2.2185	-2.39631
H	0.3302	2.81999	-1.24925
C	1.14181	1.81785	-2.93261
H	0.88097	1.04721	-3.67005

H	1.2408	2.7674	-3.47812
C	3.41626	1.19192	-3.21187
H	3.69762	2.08614	-3.78644
H	3.08941	0.41554	-3.91885
H	-1.22634	-0.95943	-1.01221
C	4.60571	0.69303	-2.42312
H	5.43381	0.46447	-3.10845
H	4.94032	1.47197	-1.72481
C	5.22928	-1.22097	-1.10756
H	6.05835	-1.37987	-1.81183
H	4.78877	-2.19635	-0.88528
C	5.76191	-0.56107	0.16019
H	6.59064	-1.17049	0.55983
H	6.19687	0.40529	-0.1041
C	4.43628	-1.56591	1.93399
H	5.13471	-1.67293	2.78179
H	4.60882	-2.42985	1.28797
C	3.00415	-1.62394	2.46111
H	2.89219	-2.49524	3.12136
H	2.76241	-0.72709	3.03611
O	1.41366	-1.77732	-1.2322
H	1.50906	-3.67608	1.49393
C	0.49834	-1.33061	-2.23188
H	0.0164	-2.19351	-2.7148
H	1.1245	-0.84055	-2.97707
C	-0.59464	-0.40084	-1.70493
H	-1.24907	-0.1343	-2.54516
C	-3.73645	-2.26292	0.92941
C	-2.6154	-1.90291	1.69162
H	-2.51443	-0.87579	2.01861
C	-1.64783	-2.84371	2.04406
H	-0.79673	-2.54116	2.64833
C	-1.76188	-4.16814	1.61098
H	-1.00569	-4.90054	1.87804
C	-2.85485	-4.53566	0.826
H	-2.95189	-5.5584	0.47249
C	-3.83207	-3.59211	0.49825
H	-4.6897	-3.89241	-0.09925
C	-4.43013	0.09365	0.04932
C	-4.47915	1.2307	0.93749
C	-4.35836	1.07897	2.3575
H	-4.22532	0.08787	2.77614
C	-4.37464	2.15474	3.23729
H	-4.25762	1.96199	4.30204
C	-4.52946	3.46809	2.77969
H	-4.53148	4.30836	3.46705
C	-4.70875	3.65294	1.40398
H	-4.88738	4.65262	1.01199
C	-4.69479	2.5823	0.51783
H	-4.91848	2.78248	-0.52136
C	-4.83595	-1.25414	0.62653
H	-5.39037	-1.087	1.55766
H	-5.55832	-1.74914	-0.03795
C	-4.09385	0.13371	-1.34355
C	-3.6079	1.28745	-2.04923
H	-3.43099	2.20622	-1.50927
C	-3.29953	1.26885	-3.40295
H	-2.94408	2.18815	-3.86583
C	-3.41395	0.10382	-4.17198
H	-3.17915	0.09821	-5.23175
C	-3.82993	-1.05715	-3.50805
H	-3.90539	-1.99495	-4.05606
C	-4.15028	-1.05053	-2.15714
H	-4.43422	-1.99247	-1.70653

5

C	-7.33345	-1.09442	1.61921
H	-8.03123	-1.22735	2.45738
H	-7.84775	-1.41091	0.70819
C	-6.91215	0.3695	1.52541
H	-7.81654	1.00251	1.55931
H	-6.32849	0.62691	2.41253
C	-5.70611	-2.03815	3.08755
H	-6.42983	-2.52239	3.7581
H	-5.49732	-1.03227	3.47181
C	-4.42001	-2.82917	3.05332
H	-4.01232	-2.92271	4.06979
H	-4.61657	-3.83743	2.666
C	-1.12357	-2.52085	-1.10234
H	-0.46816	-2.41357	-0.22738
H	-0.47139	-2.49984	-1.98842
C	-1.88638	-3.83495	-1.07943
H	-2.44068	-3.90166	-2.01684
H	-1.15981	-4.66764	-1.06934
C	-1.50551	-0.16534	-1.01901
H	-1.0138	0.14361	-1.95072
H	-0.76345	-0.15426	-0.21044
C	-2.63873	0.77099	-0.69398
H	-2.29075	1.80163	-0.63999
H	-3.39054	0.71574	-1.48745
C	-2.21644	-2.65379	2.09347
H	-1.76235	-2.78178	3.08757
H	-1.65892	-1.88637	1.55512
C	-2.18348	-3.98921	1.34506
H	-1.13645	-4.31284	1.2565
H	-2.67414	-4.75323	1.9547
C	-6.13912	0.46982	-2.13153
H	-6.78206	0.62689	-3.00885
H	-5.26467	1.11461	-2.24308
C	-6.91197	0.81524	-0.86267
H	-7.30202	1.84396	-0.95645
H	-7.78728	0.16466	-0.78856
C	-6.64482	-1.84927	-2.37136
H	-7.19512	-1.61226	-3.29281
H	-7.36568	-1.89011	-1.54404
C	-5.98125	-3.19486	-2.52411
H	-6.74415	-3.945	-2.77709
H	-5.24367	-3.16236	-3.33876
C	-3.89852	1.36077	1.25948
H	-3.25404	2.2376	1.39582
H	-4.10694	0.9309	2.24316
C	-5.18041	1.7749	0.54855
H	-5.66152	2.58981	1.11633
H	-4.91135	2.19969	-0.42061
C	-4.71573	-4.81053	-1.36008
H	-4.15004	-4.90354	-2.29667
H	-5.47722	-5.60357	-1.36471
C	-3.81941	-5.00096	-0.14826
H	-4.45286	-5.01867	0.74277
H	-3.35044	-5.99846	-0.23459
N	-6.09545	0.6433	0.3408
N	-2.83677	-3.92759	0.0307
O	-6.22133	-1.97924	1.76036
O	-2.07082	-1.4705	-1.14548
O	-3.52578	-2.11586	2.213
O	-5.64125	-0.87055	-2.12854
O	-3.18881	0.34534	0.55614
O	-5.33513	-3.53172	-1.30222
Na	-4.38185	-1.6359	-0.00768
C	5.94597	0.77973	0.61096
H	5.2774	1.20612	-0.13742
H	6.9097	1.30699	0.57155
C	5.33429	0.91136	2.00258

H	5.96395	0.39286	2.73087
H	5.34831	1.97759	2.2884
C	7.19111	-1.24387	0.89373
H	7.06407	-1.19044	1.98197
H	8.15491	-0.78154	0.63631
C	7.19245	-2.69019	0.46712
H	7.28843	-2.74938	-0.62402
H	8.04936	-3.20901	0.91904
C	2.10354	-4.30183	-1.07365
H	2.05213	-3.64975	-1.94641
H	1.19988	-4.92674	-1.04082
C	3.33773	-5.19453	-1.1507
H	3.44769	-5.7373	-0.20793
H	3.16349	-5.96163	-1.92642
C	1.86632	-4.05527	1.29758
H	2.74547	-4.61912	1.63615
H	1.02648	-4.75835	1.21073
C	1.53357	-2.98508	2.30828
H	0.67683	-2.39215	1.95712
H	1.25566	-3.45451	3.26418
C	5.8965	-4.68619	0.66598
H	5.02734	-5.03079	1.23208
H	6.78562	-5.18502	1.07788
C	5.75133	-5.03512	-0.81359
H	6.62648	-4.66784	-1.35488
H	5.76646	-6.13466	-0.91574
C	2.13816	0.64632	0.38951
H	1.53207	1.43234	-0.0654
H	1.46519	-0.13562	0.7674
C	2.97814	1.25873	1.49572
H	3.48745	2.12029	1.06265
H	2.31362	1.64297	2.27639
C	2.50915	0.06193	-1.89917
H	2.26195	1.07508	-2.23038
H	1.60498	-0.56467	-1.9507
C	3.587	-0.52536	-2.77413
H	3.29113	-0.48233	-3.83184
H	4.51147	0.0527	-2.64843
C	2.41272	-1.05445	3.34273
H	2.20143	-1.42829	4.35536
H	1.53123	-0.49442	3.0052
C	3.63707	-0.16109	3.37919
H	4.48096	-0.74287	3.76459
H	3.43384	0.65176	4.10015
C	4.90114	-2.5173	-2.95055
H	5.82395	-2.17833	-2.45668
H	4.97489	-2.26634	-4.01873
C	4.70969	-4.0191	-2.79973
H	3.80964	-4.29785	-3.35738
H	5.54919	-4.53827	-3.283
N	3.99046	0.33763	2.05372
N	4.55832	-4.43223	-1.39992
O	6.12697	-0.58692	0.21868
O	2.11286	-3.41859	0.04911
O	5.97118	-3.28191	0.90498
O	3.02436	0.08049	-0.57593
O	2.66619	-2.14999	2.46298
O	3.77752	-1.87803	-2.36348
Na	4.0273	-1.85078	0.25433
C	0.36781	3.9956	-0.99034
C	0.4966	4.58116	0.39772
C	1.58645	3.70096	-1.6953
C	1.72206	3.47702	-3.10903
H	0.8595	3.58961	-3.75164
C	2.94399	3.19291	-3.71137
H	2.96884	3.03732	-4.78922
C	4.13162	3.12045	-2.97331
H	5.08122	2.89252	-3.44806
C	4.0495	3.41632	-1.60252
H	4.95581	3.42857	-0.99891
C	2.84005	3.71027	-0.99494
H	2.81345	3.96149	0.05855
C	-0.94816	3.84367	-1.53903
C	-2.07815	4.42593	-0.86612
H	-1.87966	4.98445	0.04186
C	-3.37706	4.31302	-1.33515
H	-4.17819	4.80127	-0.78205
C	-3.68031	3.59097	-2.5032
H	-4.69522	3.53115	-2.88492
C	-2.61331	2.96123	-3.1581
H	-2.80665	2.35494	-4.04235
C	-1.30561	3.06823	-2.69868
H	-0.54481	2.49628	-3.21238
C	0.76359	5.99241	0.5025
C	0.56319	6.78349	1.68212
H	0.16527	6.31187	2.57237
C	0.79104	8.15417	1.71589
H	0.60212	8.69242	2.64419
C	1.23366	8.85339	0.58597
H	1.42059	9.92282	0.62011
C	1.40726	8.11845	-0.59593
H	1.73052	8.62682	-1.50363
C	1.17533	6.75114	-0.64343
H	1.30461	6.22468	-1.58119
C	0.33137	3.71268	1.52627
C	-0.32451	2.4432	1.38567
H	-0.68553	2.18623	0.40247
C	-0.49783	1.55024	2.43236
H	-1.03705	0.62447	2.23951
C	-0.00738	1.82927	3.71856
H	-0.1502	1.13709	4.54321
C	0.67259	3.04541	3.89285
H	1.10345	3.28552	4.86425
C	0.83483	3.95181	2.85212
H	1.42078	4.84349	3.0403

6

Na	9.09835	0.05757	0.02638
O	7.74286	-0.872	-1.7886
O	9.91133	-1.91217	1.47187
O	11.19049	0.92234	-1.03817
O	8.20278	1.84573	-1.50693
O	7.34878	-0.93547	1.31204
O	10.65013	1.18103	1.6688
N	10.47708	-1.91978	-1.43551
N	7.8155	1.97508	1.43679
C	9.60979	-2.24384	-2.57492
H	9.83655	-3.24023	-2.99234
H	9.80405	-1.5258	-3.37547
C	8.13101	-2.17541	-2.20356
H	7.5174	-2.50596	-3.05284
H	7.91064	-2.8329	-1.35901
C	10.67486	-3.07484	-0.55359
H	11.48722	-3.73159	-0.91167
H	9.76538	-3.68001	-0.56883
C	10.96769	-2.6643	0.88743
H	11.17892	-3.56085	1.48757
H	11.84889	-2.01876	0.93545
C	11.74777	-1.32443	-1.86025
H	12.1691	-1.83625	-2.74319
H	12.47823	-1.45223	-1.05766
C	11.61123	0.16408	-2.16843

H	12.56474	0.55157	-2.55384
H	10.84844	0.33465	-2.93234
C	7.1214	2.83715	0.47188
H	6.88079	3.82382	0.90786
H	6.16191	2.37575	0.22058
C	7.93784	3.05433	-0.79811
H	7.41552	3.7685	-1.45163
H	8.92119	3.47507	-0.56748
C	6.85847	1.25057	2.29483
H	6.0096	1.88804	2.58463
H	7.37303	0.96388	3.21683
C	6.30643	0.01116	1.61017
H	5.52826	-0.45733	2.21958
H	5.84371	0.25659	0.6552
C	8.80784	2.71425	2.2186
H	8.3536	3.23472	3.08104
H	9.24086	3.49463	1.58699
C	9.93059	1.81377	2.72633
H	10.61792	2.39348	3.35846
H	9.52815	0.99701	3.3304
C	7.47104	0.03186	-2.85215
H	6.64179	-0.34387	-3.46786
H	8.35189	0.15874	-3.49748
C	7.0802	1.3541	-2.23959
H	6.81038	2.0625	-3.03761
H	6.20959	1.20843	-1.59093
C	8.80337	-2.6931	1.91075
H	9.11147	-3.37394	2.71728
H	8.39454	-3.29394	1.08817
C	7.73938	-1.74113	2.40589
H	6.88271	-2.3111	2.79328
H	8.14547	-1.13513	3.2296
C	12.21957	1.21386	-0.10155
H	13.019	1.79833	-0.57883
H	12.66082	0.29431	0.30586
C	11.60033	2.01428	1.02009
H	12.38116	2.3315	1.72635
H	11.12545	2.91299	0.60471
Na	-9.10188	-0.08829	0.02461
O	-7.74164	0.84452	1.83122
O	-9.92423	1.87928	-1.41508
O	-11.18681	-0.95541	1.10523
O	-8.19295	-1.87732	1.55612
O	-7.36189	0.90302	-1.27392
O	-10.66612	-1.22114	-1.60539
N	-10.47671	1.88845	1.4959
N	-7.82865	-2.00828	-1.3904
C	-9.60319	2.21474	2.62989
H	-9.82889	3.21109	3.04797
H	-9.79132	1.49682	3.43203
C	-8.12686	2.14767	2.24916
H	-7.50767	2.47812	3.09437
H	-7.91246	2.80564	1.40353
C	-10.68145	3.04186	0.6135
H	-11.49403	3.69687	0.97427
H	-9.77362	3.6496	0.62406
C	-10.97974	2.62892	-0.8258
H	-11.19645	3.52436	-1.42569
H	-11.85932	1.98081	-0.8689
C	-11.74352	1.29087	1.9286
H	-12.16098	1.80273	2.81338
H	-12.47887	1.41598	1.13006
C	-11.60142	-0.19692	2.23764
H	-12.55127	-0.5863	2.63009
H	-10.83288	-0.36481	2.99635
C	-7.12242	-2.86548	-0.43
H	-6.87943	-3.85121	-0.86675
H	-6.16418	-2.39734	-0.18602
C	-7.92801	-3.08535	0.84658
H	-7.39735	-3.7965	1.49665
H	-8.91095	-3.51101	0.62358
C	-6.88157	-1.28325	-2.25849
H	-6.03763	-1.92274	-2.55889
H	-7.40627	-0.99618	-3.17463
C	-6.32225	-0.04383	-1.57983
H	-5.54942	0.42389	-2.19605
H	-5.85251	-0.28686	-0.62793
C	-8.82475	-2.75305	-2.16205
H	-8.37531	-3.27574	-3.0257
H	-9.25146	-3.53196	-1.52439
C	-9.95305	-1.85697	-2.66539
H	-10.64369	-2.44098	-3.28992
H	-9.55679	-1.04208	-3.27604
C	-7.45398	-0.05787	2.89169
H	-6.61888	0.32121	3.4973
H	-8.32665	-0.18743	3.54762
C	-7.065	-1.37954	2.27629
H	-6.7835	-2.08553	3.07235
H	-6.20238	-1.22986	1.6181
C	-8.81929	2.66293	-1.8573
H	-9.1327	3.34648	-2.65949
H	-8.40675	3.26106	-1.03466
C	-7.75629	1.71458	-2.36169
H	-6.90152	2.28825	-2.74784
H	-8.16437	1.11307	-3.18788
C	-12.2222	-1.25079	0.17686
H	-13.01746	-1.83492	0.66146
H	-12.66753	-0.33294	-0.22995
C	-11.61008	-2.05371	-0.94679
H	-12.39561	-2.37453	-1.64616
H	-11.13058	-2.95022	-0.53194
O	0.17668	1.44174	-0.7672
C	0.38819	1.50311	-2.20763
H	1.39399	1.11898	-2.41275
H	-0.37078	0.87205	-2.67715
C	0.25544	2.97802	-2.56957
H	-0.80006	3.22051	-2.72847
C	0.7791	3.66784	-1.30253
H	0.45693	4.70797	-1.20853
C	0.20944	2.78221	-0.20079
H	0.84031	2.74616	0.69105
H	-0.81822	3.06048	0.06166
Na	1.66788	0.02112	0.30268
C	3.84741	1.26697	-0.57053
H	4.00715	1.99158	-1.37129
C	3.93954	-0.07544	-1.02513
H	4.1799	-0.14158	-2.08816
C	3.79993	-1.35057	-0.4185
H	3.96457	-2.16696	-1.12472
C	3.47939	-1.81328	0.88281
H	3.44303	-2.90316	0.93931
C	3.14486	-1.19586	2.11165
H	2.89843	-1.92025	2.89053
C	3.00837	0.14445	2.5541
H	2.6961	0.2115	3.59852
C	3.18613	1.41999	1.95772
H	2.98586	2.23739	2.65382
C	3.54467	1.88456	0.66752
H	3.54891	2.97416	0.60138
Na	-1.7035	0.17288	-0.24576
O	-0.17217	-1.35008	0.61646
C	-0.3793	-1.68182	2.0185
H	-1.41535	-1.42821	2.27043
H	0.32127	-1.08167	2.60449

C	-0.11462	-3.17948	2.11903
H	-0.64711	-3.64066	2.95477
C	-0.58239	-3.67931	0.74566
H	-0.17031	-4.65461	0.47429
C	-0.09776	-2.56858	-0.17839
H	-0.73679	-2.42787	-1.05349
H	0.94825	-2.70867	-0.47495
C	-3.81552	-1.31482	0.47092
H	-3.93424	-2.12432	1.19407
C	-3.97527	-0.03185	1.05909
H	-4.20176	-0.08533	2.12578
C	-3.92086	1.30402	0.58336
H	-4.13223	2.03379	1.36749
C	-3.64615	1.91379	-0.66818
H	-3.69919	3.00358	-0.6201
C	-3.27168	1.44412	-1.94992
H	-3.08453	2.25592	-2.65593
C	-3.04247	0.16602	-2.51859
H	-2.71868	0.2233	-3.56013
C	-3.14174	-1.17096	-2.05365
H	-2.88594	-1.90022	-2.82565
C	-3.48098	-1.78421	-0.82208
H	-3.42498	-2.87351	-0.8677
H	0.95955	-3.34908	2.24129
H	-1.67483	-3.72883	0.70824
H	1.87206	3.62609	-1.26878
H	0.81837	3.23667	-3.46993

7

C	0.91135	-2.54318	-0.73474
H	1.35436	-2.53144	0.26431
H	1.65631	-2.99778	-1.3949
C	-0.39303	-3.32254	-0.76614
H	-0.7875	-3.3072	-1.78504
H	-0.18206	-4.38046	-0.5318
C	0.75018	-0.94086	-2.51454
H	-0.00475	-1.56046	-3.01313
H	1.75031	-1.18458	-2.89147
C	0.46375	0.51962	-2.76361
H	1.23195	1.13564	-2.27771
H	0.51073	0.71554	-3.84394
C	1.07222	2.44194	1.00553
H	1.63568	2.47153	0.06992
H	1.74456	2.83723	1.77281
C	-0.2036	3.26306	0.94274
H	-0.69688	3.21757	1.91695
H	0.06415	4.32192	0.77783
C	0.76712	0.7437	2.66527
H	0.09636	1.43375	3.19105
H	1.78323	0.83061	3.07001
C	0.27617	-0.67335	2.83443
H	0.96182	-1.36888	2.33417
H	0.26681	-0.92339	3.90445
C	-1.16044	-3.09718	1.53735
H	-0.08514	-3.2136	1.68802
H	-1.61376	-4.06498	1.81009
C	-1.67921	-2.0207	2.48658
H	-2.74279	-1.83312	2.31763
H	-1.5544	-2.35173	3.5266
C	-1.27712	2.14814	-2.47285
H	-2.36866	2.11166	-2.42351
H	-0.99858	2.48602	-3.47997
C	-0.71571	3.11511	-1.43457
H	0.37479	3.076	-1.47537
H	-0.99987	4.14482	-1.71148
C	-3.26419	-2.25964	-1.43985
H	-2.56963	-2.28246	-2.28294
H	-4.23356	-2.63502	-1.79482
C	-2.76142	-3.12953	-0.29251
H	-3.43667	-3.01567	0.55915
H	-2.81809	-4.19073	-0.59092
C	-4.55059	-0.58055	-0.333
H	-4.61189	-1.17956	0.58526
H	-5.44504	-0.78435	-0.93806
C	-4.5002	0.88291	0.0258
H	-4.40309	1.48048	-0.89034
H	-5.4316	1.17436	0.53093
C	-3.19952	2.43825	1.28501
H	-2.6011	2.39779	2.19797
H	-4.16582	2.89505	1.53794
C	-2.50382	3.26513	0.20864
H	-3.09205	3.21497	-0.71058
H	-2.4998	4.32587	0.51402
C	4.20602	-0.65295	-1.80238
C	4.24766	-1.71483	-0.86379
C	4.24309	-1.80565	0.55053
C	4.26291	-0.86902	1.61821
C	4.31978	0.54566	1.70362
C	4.31936	1.60622	0.76364
C	4.22363	1.69861	-0.64755
C	4.16611	0.76129	-1.71436
N	-1.40717	-2.75485	0.13065
N	-1.14762	2.77668	-0.07158
Na	-1.2552	0.02896	0.00575
Na	2.42998	-0.04163	0.01466
O	0.71727	-1.16823	-1.09965
O	0.79292	1.05675	1.26862
O	-1.04067	-0.75838	2.29488
O	-0.84236	0.80519	-2.26647
O	-3.37268	-0.88651	-1.06938
O	-3.38572	1.08024	0.88834
H	4.21602	-2.83903	0.90315
H	4.09338	1.24229	-2.69194
H	4.22171	-2.69553	-1.34371
H	4.18853	2.73235	-0.99839
H	4.32916	2.58697	1.24373
H	4.16481	-1.00861	-2.83427
H	4.24491	-1.34947	2.5987
H	4.34117	0.90157	2.73603

THF

H	-1.57637	-1.10822	0.47366
O	0.	0.	1.25435
C	0.47804	1.07292	0.42724
C	0.	0.76723	-0.99463
C	0.	-0.76723	-0.99463
C	-0.47804	-1.07292	0.42724
H	0.09016	2.01959	0.81867
H	1.57637	1.10822	0.47366
H	0.64686	1.2047	-1.75908
H	-1.01606	1.14717	-1.14631
H	1.01606	-1.14717	-1.14631
H	-0.64686	-1.2047	-1.75908
H	-0.09016	-2.01959	0.81867

COT

C	0.56282	0.47455	-1.58002
C	0.87007	1.37214	-0.63138
C	0.15363	1.61742	0.6313
C	-0.15363	0.71994	1.5801

H	1.15567	0.48591	-2.49523	C	-0.87007	-1.37214	-0.63138
H	1.69355	2.05751	-0.83423	H	-1.15567	-0.48591	-2.49523
H	-0.07681	2.66362	0.83429	C	-0.15363	-1.61742	0.6313
H	-0.61469	1.09244	2.49522	H	0.61469	-1.09244	2.49522
C	-0.56282	-0.47455	-1.58002	H	-1.69355	-2.05751	-0.83423
C	0.15363	-0.71994	1.5801	H	0.07681	-2.66362	0.83429

Section 3. References

[1] Clark R. C.; Reid J. S. The analytical calculation of absorption in multifaceted crystals, *Acta Crystallogr., Sect. A* **1995**, *51*, 887. DOI: 10.1107/S0108767395007367

[2] *CrysAlisPro*, Rigaku Oxford Diffraction, Tokyo, Japan.

[3] Sheldrick G. M. Crystal structure refinement with SHELXL. *Acta Crystallogr., Sect. A: Found. Crystallogr.* **2015**, *71,* 3-8. DOI: 10.1107/S2053229614024218

[4] Sheldrick G. M. A short history of SHELX. *Acta Crystallogr., Sect. A: Found. Crystallogr.* **2008**, *64*, 112-122. DOI: 10.1107/S0108767307043930

[5] Dolomanov O. V.; Bourhis L. J.; Gildea R. J.; Howard J. A. K.; Puschmann H. OLEX2: a complete structure solution, refinement and analysis program. *J. Appl. Cryst.* **2009**, *42*, 339-341. DOI: 10.1107/S0021889808042726

[7] Becke, A. D. Density-functional thermochemistry. III. The role of exact exchange. *J. Chem. Phys.* **1993**, *98*, 5648-5652. DOI: 10.1063/1.464913

[8] Lee, C. T.; Yang, W. T. ; Parr, R. G. Development of the Colle-Salvetti correlation-energy formula into a functional of the electron density. *Phys. Rev. B* **1988**, *37*, 785. DOI: 10.1103/PhysRevB.37.785

[9] Grimme, S.; Ehrlich, S.; Goerigk, L. Effect of the damping function in dispersion corrected density functional theory. *J. Comput. Chem.* **2011**, *32*, 1456-1465. DOI: 10.1002/jcc.21759

[10] Glendening E. D.; Reed A. E.; Carpenter J. E.; Weinhold F. *NBO Version 3.1*, TCI, University of Wisconsin, Madison, 1998.

[11] Gaussian 16, Revision C.02-AVX2, Frisch M. J.; Trucks G. W.; Schlegel H. B.; Scuseria G. E.; Robb M. A.; Cheeseman J. R.; Scalmani G.; Barone V.; Petersson G. A.; Nakatsuji H.; Li X.; Caricato M.; Marenich A. V.; Bloino J.; Janesko B. G.; Gomperts R.; Mennucci B.; Hratchian H. P.; Ortiz J. V.; Izmaylov A. F.; Sonnenberg J. L.; Williams-Young D.; Ding F.; Lipparini F.; Egidi F.; Goings J.; Peng B.; Petrone A.; Henderson T.; Ranasinghe D.; Zakrzewski V. G.; Gao J.; Rega N.; Zheng G.; Liang W.; Hada M.; Ehara M.; Toyota K.; Fukuda R.; Hasegawa J.; Ishida M.; Nakajima T.; Honda Y.; Kitao O.; Nakai H.; Vreven T.; Throssell K.; Montgomery J. A.; Peralta Jr.; J. E.; Ogliaro F.; Bearpark M. J.; Heyd J. J.; Brothers E. N.; Kudin K. N.; Staroverov V. N.; Keith T. A.; Kobayashi R.; Normand J.; Raghavachari K.; Rendell A. P.; Burant J. C.; Iyengar S. S.; Tomasi J.; Cossi M.; Millam J. M.; Klene M.; Adamo C.; Cammi R.; Ochterski J. W.; Martin R. L.; Morokuma K.; Farkas O.; Foresman J. B.; Fox D. J.; Gaussian, Inc. Wallingford CT, 2016.

[12]Chemcraft - graphical software for visualization of quantum chemistry computations. Version 1.8, build 682. https://www.chemcraftprog.com
